# Human urine stem cells protect against cyclophosphamide-induced premature ovarian failure by inhibiting SLC1A4-mediated outflux of intracellular serine in ovarian granulosa cells

**DOI:** 10.1186/s11658-025-00701-1

**Published:** 2025-02-19

**Authors:** Hao-Cheng Gu, Ling-Fang Wang, Yu-Wei Zhang, You-Qiong Zhuo, Zhou-Hang Zhang, Xing-Yu Wei, Quan-Wen Liu, Ke-Yu Deng, Hong-Bo Xin

**Affiliations:** 1 The National Engineering Research Center for Bioengineering Drugs and the Technologies, Institute of Translational Medicine, Jiangxi Medical College, Nanchang, 330031 People’s Republic of China; 2 School of Life and Science, Nanchang University, Nanchang, 330031 People’s Republic of China; 3 School of Food Science and Technology, Nanchang University, Nanchang, 330031 People’s Republic of China

**Keywords:** Premature ovarian failure, Human urine-derived stem cells, miR-27b-3p, SLC1A4, Ovarian granulosa cells, Cyclophosphamide

## Abstract

**Background:**

Cyclophosphamide (CTX) is the first-line medication for the treatment of breast cancer, although it potentially leads to severe ovarian dysfunction and even premature ovarian failure (POF). However, the mechanism of CTX-induced POF remains unclear. Mesenchymal stem cell-based therapy has been wildly used for treating numerous diseases. Therefore, our study aims to elucidate the underlying mechanism of CTX-induced POF and to explore the therapeutic effect of human urine stem cells (hUSCs) in POF.

**Methods:**

CTX-induced POF or ovarian granulosa cell (GCs) apoptosis were treated with hUSCs and their exosomes in vitro and in vivo. Morphological, histological, and functional alternations were examined using multiple approaches. The effector molecules of hUSC-derived exosomes (hUSC-Exo) were determined by differential expression analysis in the ovaries. The target genes of miRNA were accessed by transcriptome sequencing in GCs, and the underlying mechanisms were further elucidated.

**Results:**

hUSCs remarkably inhibited CTX-induced apoptosis and promoted the proliferation of GCs, respectively. In addition, we observed that miR-27b-3p was highly expressed in hUSC-Exo and markedly suppressed CTX-induced GC apoptosis by specifically inhibiting the expression of SLC1A4, a serine transporter, in ovarian GCs, which, in turn, elevated the concentration of the intracellular serine by inhibiting the outflux of cellular serine. More importantly, the knockdown of SLC1A4 or simple supplementation of serine suppressed CTX-induced apoptosis of GCs. Finally, we demonstrated that CTX-induced apoptosis of ovarian GCs was essential for POF by reducing the intracellular serine concentration via elevating the expression of SLC1A4, whereas hUSCs protected against CTX-induced POF via miR-27b-3p/SLC1A4/serine axis-mediated activation of the PI3K/AKT/mTOR signaling pathway.

**Conclusions:**

Our study suggests that hUSC-based cell therapy or simple supplementation of serine may provide an efficient therapeutic approach for the prevention and treatment of CTX-induced POF clinically.

**Graphical abstract:**

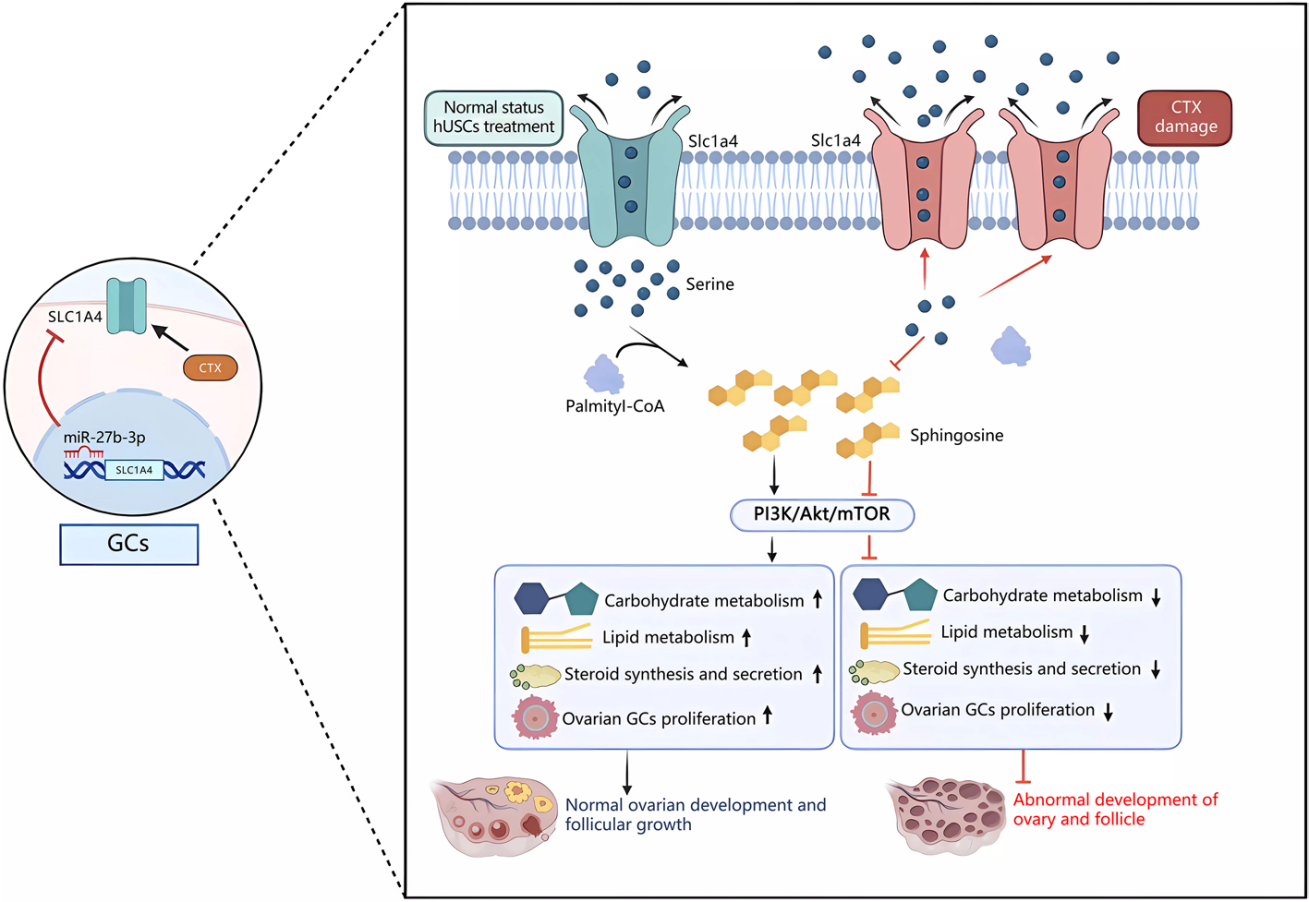

**Supplementary Information:**

The online version contains supplementary material available at 10.1186/s11658-025-00701-1.

## Introduction

Cyclophosphamide (CTX)-induced premature ovarian failure (POF) is the most common side effect of chemotherapy in breast cancer, with an incidence rate as high as 40% [1]. As a functional defect of ovaries, POF seriously affects the physical and mental health of women, accompanied by many serious consequences such as high blood pressure, osteoporosis, infertility, cardiovascular disease, and psychological disorders [2]. Ovarian granulosa cells (GCs) are the main functional cells that secrete sex hormones, including estrogen for maturation of oocytes in ovaries [3]. Apoptosis of GCs in follicles results in follicular atrophy and atresia and eventually a lack of ovarian functions [4]. However, there is no effective method to protect against CTX-induced ovarian damage clinically owing to the unclear mechanism of CTX-induced POF. Therefore, seeking a new therapeutic approach to delay or treat POF induced by CTX is essential and urgent.

Mesenchymal stem cells (MSCs) have been widely used in therapeutic approaches for a variety of diseases owing to their pluripotency, low immunogenicity, lack of tumorigenicity, anti-inflammatory function, and high proliferation [5]. Numerous studies have shown that MSCs from several different sources could restore ovarian function in POF mice [6–9]. Human urine-derived stem cells (hUSCs) are a novel subpopulation of stem cells that have some advantages such as high self-renewal, proliferation, multidirectional differentiation abilities, and they are easily obtained from urine [10]. Studies have shown that hUSCs have therapeutic potential for many diseases such as bladder repair [11], erectile dysfunction [12], diabetes [13], cartilage defects [14], skin damage [14], and acute kidney injury [15]. However, the roles and mechanisms of hUSCs in POF have not been explored.

SLC1A4, a neutral amino acid transporter, is responsible for the transportation of amino acids, such as cysteine, serine, and alanine, in mammalian cells [16]. Serine plays a crucial role in cellular growth, organ development, and various biochemical processes involved in the proliferation of cells, neurons, stem cells, muscle cells, and immune cells [17]. Studies have shown that the dysregulated expression of SLC1A4 is accompanied by an abnormity of the intracellular serine levels of nerve cells in many neurological disorders, thereby impacting the normal growth and development of the nervous system [18].

In the present study, we aimed to explore the mechanism of CTX-induced POF and the therapeutic effect of hUSCs on CTX-induced POF.

## Materials and methods

### Isolation, culture, and expansion of hUSCs

The isolation and culture of hUSCs were performed according to a previously reported protocol [19]. Briefly, 100 mL of urine was collected from each healthy male participant, with a total of 10 persons aged between 20 and 30 years. Each sample was centrifuged at 400 g for 10 min, and the pellets were resuspended with 10 mL of phosphate buffered saline (PBS) solution, followed by a second centrifugation at 400 g for 10 min. Then, the supernatant was removed as completely as possible, and the cells in the pellets were resuspended with a 1 mL hUSC culture medium containing KnockOut Serum Replacement (KSFM), 22% High Glucose-Dulbecco’s Modified Eagle Medium (H-DMEM), 22% Dulbecco’s Modified Eagle Medium/Nutrient Mixture F-12 (DMEM/F12), 10% fetal bovine serum (FBS), 1% insulin-transferrin-selenium (insulin-transferrin-Se), 100 U/mL penicillin and streptomycin (all from Gbico), 10 mol/L cholera toxin and adenine, 2 × 10^–9^ mol/L 3,3′,5-Triiodo-L-thyronine, hydrocortisone (all from Sigma), and 10 ng/mL epidermal growth factor (EGF) (Peprotech, USA). They were cultured in 12-well plates in a 5% CO_2_ incubator. The medium was changed daily for 3 days and then it was replaced every 3 days. hUSC colonies typically emerged after 7–10 days of culture, designated as passage 0 (P0). The cells were transferred to a 10 cm dish from a 6-well plate when they reached 80% confluence (P2) for further expansion. Usually, hUSCs from passages 3–5 (P3-P5) were utilized for further experiments.

### Preparation of conditioned media and exosomes

hUSCs and DFL (deep fiber layer) cells were cultured in an H-DMEM medium (without serum) for 48 h and then their supernatant was collected and centrifuged at 5000 g for 1 h at 4 ℃ to obtain hUSC-conditioned medium (hUSC-CM) concentrated to 10-fold. For preparing exosomes, the supernatant was centrifuged at 1000 g, 3000 g, and 10,000 g for 10 min at 4 ℃, respectively, followed by a final centrifugation at 100,000 g for 1 h at 4 ℃ using an ultra-high-speed centrifuge (Beckman, USA). The concentrations of hUSC-Exo and DFL-Exo were adjusted to 30 μg/mL, and the exosomes were identified by morphology using electron microscopy, particle size examination with nanoparticle tracking analysis, and specific marker detection with Western blot (WB) analysis.

### Reverse transcription-polymerase chain reaction (RT-PCR) and quantitative reverse transcription-polymerase chain reaction (qRT-PCR)

Total RNA was isolated from ovarian granulosa cells (GCs) using the TRIzol reagent (Thermo Fisher, USA), and the RNA was reversely transcribed into cDNA using M-MLV reverse transcriptase (Promega, Shanghai, China). The primers for the target genes are provided in Supplementary Table 1. Polymerase chain reactions (PCR) were conducted in a PCR thermal cycler (Thermo Hybaid, USA). PCR products were subjected to electrophoresis on a 1.0% (mass/volume) agarose gel containing 0.5 μg/mL ethidium bromide for nucleic acid visualization under ultraviolet (UV) light. Human GAPDH served as an internal control. Quantitative PCR was performed using the ABI-ViiA7 PCR machine. The primers for qPCR are listed in Supplementary Table 2. The housekeeping gene, GAPDH, was used to normalize the gene expression.

### Flow cytometry analysis

hUSCs were characterized by flow cytometry as follows: the cells were washed and resuspended in PBS (1 × 10_6_cells/mL) and were incubated with antibodies targeting MSC markers (CD90-FITC (561969), CD29-APC (561749), CD73-PE (561,014), and CD105-PE (562380)), hematopoietic cell markers (CD34-PE (560941) and CD45-FITC (560976)), major histocompatibility antigens (HLA-ABC-PE (560168) and HLA-DR-FITC (347363)), and costimulatory molecules (CD80-FITC (557226), CD86-PE (560957), and CD40-FITC (555588)) (all from BD Biosciences) for 30 min. Then, the cells were washed twice with PBS and resuspended in 400 μL of PBS for flow cytometry analysis (Beckman, USA). The results were analyzed using Flowjo version 10.8.1 software. The cells without treatment were used as a negative control, and the region with the highest cell concentration was designated as the P1 area. Subsequently, the position of the cell group in the P1 area was employed as the negative region for the gate, specifically the quadrant 1 lower left (Q1-LL) area depicted in the figure.

### Soft agar tumorigenicity test

The bottom layer of the soft agar (0.6%) was prepared in 6-well plates, and the hUSCs were resuspended in 0.3% soft agar at a concentration of 1 × 10^3^cells/well and then plated onto the plates with 0.6% soft agar. They were incubated at 37 °C with 5% CO_2_ for 30 days. 4T1 cells were used as the control. The colonies were observed and imaged by phase contrast microscopy.

### In vivo tumorigenicity test

hUSCs and breast cancer cells (4T1, CBP60352, Nanjing Cobioer Biosciences Co., Ltd.) were suspended in 100 µL of PBS at a concentration of 1 × 10^6^ cells and injected into the breast pads of NOD-SCID mice under anesthetization; these mice were purchased from Changsha SLAC Laboratory Animal Company (Changsha, China, http://www.hnsja.com/) 4T1 cells served as a positive control. Tumor formation was monitored daily for 20 weeks.

### Animal models

C57BL/6 female mice, which were 8 weeks old, were purchased from Changsha SLAC Laboratory Animal Company (Changsha, China, http://www.hnsja.com/) and maintained on 12-h light/dark cycles with food and water available ad libitum at the Laboratory Animal Center of Institute of Translational Medicine of Nanchang University. A total of 30 mice were injected intraperitoneally with CTX (50 mg/kg; MCE, USA) for 15 days to establish POF models. All animal procedures described here were reviewed and approved by the Animal Care and Use Committee of Nanchang University.

### Lentiviral transduction of hUSCs

hUSCs were seeded in a 6-well plate with 2 mL of complete medium and incubated at 37 °C with 5% CO_2_. Then, lentiviral transduction was performed once the cell density reached 30–40%. Briefly, the cultured medium of the cells was replaced and cultured with 1 mL of the mixture of complete medium containing polybrene (8 μg/mL) for 24 h. Then, the appropriate volume of virus was used to infect hUSCs. Following the infection, the hUSCs were incubated at 37 ℃ with 5% CO_2_ overnight and then additionally incubated at 37 ℃ with 5% CO_2_ overnight after replacing the culture medium with 1 mL of complete medium. Finally, the transfection efficiency was determined by detecting immunofluorescence of green fluorescent protein (GFP) in hUSCs.

### Cell transplantation

When hUSC confluence reached 85%, the cells were infected with lentivirus labeled with enhanced green fluorescent protein (EGFP) and luciferase at a multiplicity of infection (MOI) rate of 10. Then, 2 days after growth, the cells were examined for EGFP expression by immunofluorescence microscope. In total, 40 mice were randomly divided into four groups with ten mice in each. Mice without the CTX injection served as a control (normal group, *n* = 10) and the remaining 30 mice were treated with CTX to establish a POF model. The mice treated with CTX were used as the model group (CTX group, *n* = 10). Subsequently, 1 week later, the modeling mice were injected with hUSCs labeled with GPF (hUSC group, *n* = 10, 1.5 × 10^6^ hUSCs) or with the concentrated hUSC-conditioned medium (hUSC-CM group, *n* = 10, 400 μL hUSC-CM) once a week for a total of 3 weeks, respectively. The ovarian tissues and serum of the mice were collected after 1 week for further experiments.

### Whole-body fluorescent imaging

After 1, 3, and 7 days of hUSC transplantation, the distribution of the stem cells was examined using whole-body fluorescent imaging system (LB983; Berthold, Germany) in mice. The mice transplanted with hUSCs were euthanized after 1 week, and the liver, heart, spleen, lungs, kidneys, pancreas, and brain were harvested and visualized using the imaging system.

### Enzyme-linked immunosorbent (ELISA) assay

Mouse blood samples were kept at room temperature for 4 h and then subsequently left overnight in a 4 °C refrigerator. They were centrifuged at 2000 g for 20 min and the supernatant was collected. Serum estradiol (E2) and follicle stimulating hormone (FSH) levels were measured using an ELISA kit (Cloud Clone Corp, China) according to the manufacturer’s protocol.

### Histopathology

Ovarian tissues were collected 1 week after the third cell transplantation, fixed in 4% paraformaldehyde for 24 h, subsequently dehydrated and embedded, and cut into 5 μm thick slices for hematoxylin and eosin staining (H&E staining). The follicles were classified and counted according to the previous study [20]. Follicles in the ovary are divided into four classifications: primordial follicles, primary follicles, secondary follicles, and atretic follicles.

### Terminal deoxynucleotidyltransferase-mediated deoxyUTP nick end labeling (TUNEL) assay

The ovaries were fixed in 4% formaldehyde for 24 h and embedded in paraffin. Then, they were cut into 5-μm-thick slices and dewaxed, and apoptosis was detected using a TUNEL kit (Beyotime, China). Five sections of each tissue were examined and analyzed in each experiment.

### Western blot analysis

Total proteins were extracted from ovarian tissues using a dedicated total protein kit. Briefly, the ovarian tissues were homogenized in lysis solution (R0010, Solarbio, China) with grinding beads (YA3031, Solarbio, China) and centrifuged at 2000 rpm for 1 min. In addition, the total proteins from GCs and hUSC-Exo were extracted by solely employing a lysis buffer to disrupt both the cells and the exosomes, thoroughly vortaxing the mixture for 30 s and repeating this process five times. The protein concentrations were determined by the bicinchoninic acid method (BCA; PC0020, Solarbio, China). The proteins were run on 10% denaturing SDS-PAGE gels, then transferred to polyvinylidene fluoride (PVDF) membranes (BioRad, USA), which were incubated with primary antibodies at 4 °C overnight. The antibodies used in the study were as follows: anti-GAPDH (1:5000, D4C6R, mouse monoclonal, CST), anti-FSHR (1:1000, CL594-22,665 rabbit monoclonal, protein technology), anti-Bcl-2 (1:1000, D55G8, rabbit monoclonal, CST), anti-P-AKT (1:1000, D9E, rabbit monoclonal, CST), anti-AKT (1:1000, 9272, rabbit monoclonal, CST), anti-P-mTOR (1:1000, D9C2, rabbit monoclonal, CST), anti-mTOR (1:1000, 7C10, rabbit monoclonal, CST), anti-P-PI3K (1:1000, E3U1H, rabbit monoclonal, CST), anti-PI3K (1:1000, 19H8,rabbit monoclonal, CST), anti-CD81 (1:1000, D3N2D, rabbit monoclonal, CST), anti-TSG101 (1:1000, E6V1X rabbit monoclonal, CST), anti-CD63 (1:1000, E1W3T rabbit monoclonal, CST), and anti-CD9 (1:1000, D8O1A rabbit monoclonal, CST). After 16 h, the blots were detected using horseradish peroxidase (HRP)-conjugated goat anti-rabbit or rabbit anti-mouse secondary antibody (Invitrogen, USA) for 1 h at room temperature. The images were quantified using the Super Signal West Pico chemiluminescence detection system.

### Immunofluorescence staining

GCs, hUSCs, or ovarian tissue were fixed with 4% paraformaldehyde and permeabilized with 0.1% Triton X-100, followed by incubation with the corresponding antibodies at 4 °C overnight. The antibodies used in the study were as follows: anti-FSHR (1:1000, CL594-22665 rabbit monoclonal, protein technology), anti-PCNA (1:200, D3H8P rabbit monoclonal, CST), anti-Oct4 (1:200, D7O5Z mouse monoclonal, CST), anti-SSEA4 (1:300, MC813, mouse monoclonal, CST), anti-Nanog (1:200, D73G4 rabbit monoclonal, CST), and anti-MAB1281 (1:300, mouse monoclonal, 3189191, EMD Millipore, USA). After washing in PBS, the cells were incubated for 1 h at 37 °C with a secondary biotinylated goat anti-rabbit immunoglobulin G (IgG) antibody (dilution 1:300). The cells were then washed in PBS and incubated for 3 min at 37 °C with DAPI dye liquor. The corresponding immunofluorescence staining was recorded with a laser confocal microscope (Olympus CKX41).

### Annexin V-propidium iodide (PI) apoptosis assay

For the apoptosis assays, the cells were collected and resuspended in 100 μL annexin V binding solution containing 5 μL annexin V-fluorescein isothiocyanate (FITC) and 5 μL propidium iodide (PI) solution (AD10, Dojindo, Japan). After incubation of 15 min at room temperature, the cells were washed with PBS, centrifuged at 1000 rpm for 5 min, and resuspended in 400 μL annexin V binding buffer. The apoptosis assays were detected and analyzed with BD Jazz.

### Live–dead staining

The live–dead staining of cells was performed using the live–dead staining kit (CA1630, Solarbio, China) with a working solution (PBS: diluent: PI: calcein AM = 900:100:1:1), which was added into the medium with the ratio of staining solution (medium = 1:2). The cells were incubated for 15–20 min at 37 °C , being kept away from light, and then detected with a fluorescent microscope.

### microRNA (miRNA) sequencing

Total RNA was extracted using the Total RNA Purification Kit (LC Sciences, Houston, USA), according to the manufacturer’s protocol. The total RNA quantity and purity were analyzed using Bioanalyzer 2100 and RNA 6000 Nano LabChip Kit (Agilent, CA, USA) with an RNA integrity number (RIN)  > 7.0. Approximately 1 μg of the total RNA was used to construct a small RNA library, according to the protocol of the TruSeq Small RNA Sample Prep Kits (Illumina, San Diego, USA). Single-end sequencing (1 × 50 base pairs (bp)) was performed by LC-BIO (Hangzhou, China) using Illumina Hiseq2500.

The differentially expressed miRNAs were identified by volcano plot analysis and heat map analysis. The volcano diagram features the abscissa represented as log2 and the ordinate as –log10. In this diagram, red dots represent significant upregulated miRNAs, blue dots denote significant downregulated miRNAs, and gray dots indicate the miRNAs without differential expression. The heat map utilizes log10 (normalized value) to depict the expression levels of miRNAs with the abscissa representing the samples and the ordinate representing the miRNAs. Various colors on the heat map correspond to different levels of miRNA expression.

### Transcriptome sequencing

Total RNAs were used for transcriptome sequencing. Briefly, mRNA was purified from total RNA using poly-T oligo-attached magnetic beads. Fragmentation was carried out using divalent cations under elevated temperature in First Strand Synthesis Reaction Buffer (5X). First strand cDNA was synthesized using a random hexamer primer and M-MuLV reverse transcriptase (RNase H–). Second strand cDNA synthesis was subsequently performed using DNA polymerase I and RNase H. Remaining overhangs were converted into blunt ends via exonuclease/polymerase activities. After the adenylation of 3′ends of DNA fragments, adaptors with a hairpin loop structure were ligated to prepare for hybridization to select cDNA fragments preferentially between 370 and 420 bp in length. The library fragments were purified using the APure XP system (Beckman Coulter, Beverly, USA). Then, PCR was performed with Phusion High-Fidelity DNA Polymerase, universal PCR primers, and Index (X) Primer. Finally, PCR products were purified using the AMPure XP system and the library quality was assessed on the Agilent Bioanalyzer 2100 system.

The clustering of the index-coded samples was performed on a cBot Cluster Generation System using the TruSeq PE Cluster Kit v3-cBot-HS (Illumia), according to the manufacturer’s instructions. After clustering, the library was sequenced by the Illumina Novaseq platform and 150 bp paired-end reads were generated.

### Overexpression of miRNA

Ectopic overexpression of miRNA was achieved by transient transfection of has-miR-27b-3p miRNA mimics (100 nM) (miR10000419-1-5, RIB BIO, China). Transfections were conducted using Lipofectamine® 2000 or Lipofectamine® 3000, following the manufacturer’s instructions. The concentrations of miRNA mimics and transfection reagents were optimized using Alexa Fluor Red Fluorescent Control (RIB BIO, China), along with subsequent miRNA-specific RT-qPCR, utilizing the TaqMan MicroRNA Reverse Transcription Kit from Thermo Fisher Scientific.

### Statistical analysis

The results were presented as means ± standard deviations (SD). The unpaired *t*-test was used for analysis between the two groups. One-way analysis of variance (ANOVA) was used to compare data among three or more groups, as indicated. Differences with a *P*-value of < 0.05 were considered statistically significant. The software used for data analysis was Prims 10.

## Results

### Identification and characteristics of hUSCs

**Fig. 1:**
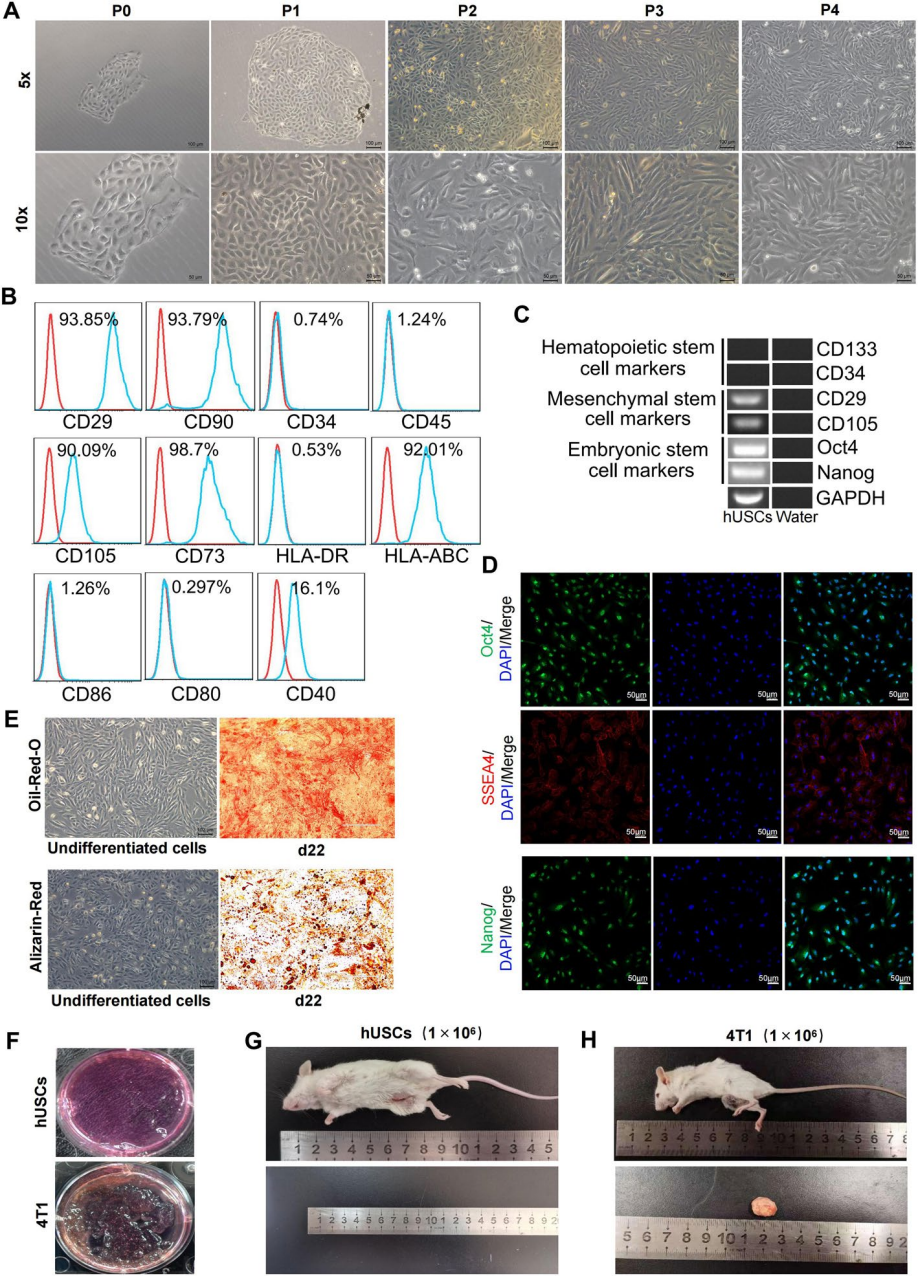
Characteristics analysis of hUSCs. **A** Morphological images of hUSCs at different culture passages (scale bar = 100 μm and 50 μm). **B** The expressions of CD29, CD90, CD105, CD73, HLA-ABC, CD34, CD45, and HLA-DR and the co-stimulatory molecules CD80, CD86, and CD40 were examined by flow cytometry analysis. **C** The expressions of Nanog, Oct4, CD105, CD29, CD34, and CD133 were analyzed by RT-PCR analysis. **D** The expressions of the embryonic stem cell surface markers Oct4, SSEA-4, and Nanog in hUSCs were identified by immunofluorescence staining. **E** The hUSCs were differentiated into mature adipocytes and osteoblasts, with osteogenic or lipogenic differentiation media for the corresponding times. Osteoblasts and adipocytes differentiated by hUSCs were identified by staining with alizarin red and oil red O, respectively. **F** In vitro tumorigenicity of hUSCs was conducted in soft agar. Numerous colonies appeared in the soft agar in the 4T1 tumor cells (1 × 10^3^/well) but not in the hUSCs, after an inoculation of 30 days. **G**, **H** In vivo tumorigenicity of hUSCs was performed in NOD-SCID mice with injection of 1 × 10^6^ cells/100 μl. Visible tumor was observed in the 4T1 cell group at the breast pads in week 2 after inoculation, and the tumor presented in the image was at week 8 after inoculation, whereas there was no tumor formation in the hUSCs group after 20 weeks of inoculation

The adherent hUSCs were obtained from the primary culture within 5 to 12 days, and afterward, the hUSCs grew rapidly, exhibiting a cobblestone-like morphology (Fig. 1A). RT-PCR and flow cytometry analysis showed that hUSCs highly expressed embryonic stem cell markers, including Oct4 and Nanog, mesenchymal stem cell markers such as CD105, CD90, CD29, and CD73, and major histocompatibility protein HLA-ABC but did not express the hematopoietic stem cell markers such as CD34, CD45, CD133, major histocompatibility protein HLA-DR, and costimulatory molecules CD80 and CD86. In addition, they had a low expression of the costimulatory factor CD40 (Fig. 1B, C). The immunofluorescence staining results showed that hUSCs expressed embryonic stem cell surface markers such as Nanog, Oct4, and SSEA-4 (Fig. 1D), suggesting that hUSCs were characterized by the stemness, with a low immunogenicity. In addition, the oil red O and alizarin red staining results showed that hUSCs had the potential for differentiating into lipocytes and osteocytes (Fig. 1E). Furthermore, the tumorigenicity assays showed that numerous clones of 4T1 cells (positive control) were formed in soft agars after being inoculated for 30 days, whereas no colonies were observed in the hUSCs group (Fig. 1F) The in vivo study showed that all five NOD/SCID mice injected with hUSCs had no tumor formation after inoculation at 20 weeks, whereas the five control mice injected with 4T1 cells formed tumors at 7–9 weeks (Fig. 1G, H). All these results indicated that hUSCs had the potential for self-renewal and multidirectional differentiation, but were less immunogenic and non-tumorigenic.

### Homing effect of hUSCs

**Fig. 2:**
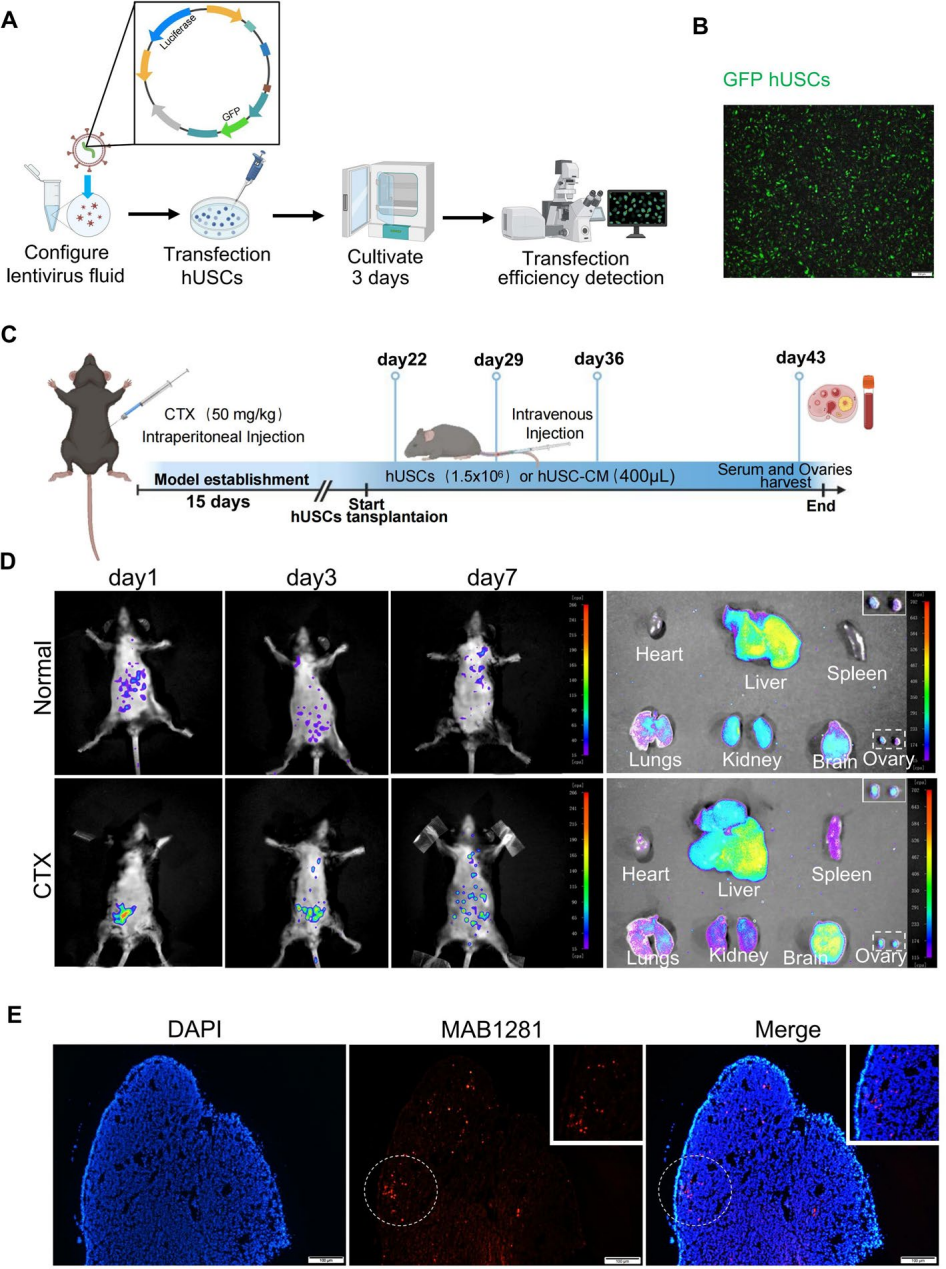
Homing of hUSCs in mouse ovaries. **A** Lentiviruses expressing GFP and luciferase genes were transfected into hUSCs. The transfection efficiency was detected by fluorescence microscopy. **B** hUSCs expressing GFP genes were observed under fluorescence microscopy. **C** A schematic diagram of the animal experimental procedure for preparing the CTX-induced POF model, as well as the cell transplantation. **D** The distribution of GFP^+^hUSCs was observed by in vivo imaging. The distribution of GFP^+^hUSCs in various organs of mice including heart, liver, spleen, lungs, kidneys, brain and ovaries was observed by in vivo imaging at the time of the sample harvest. **E** The hUSCs were stained with MAB1281, and the distribution of hUSCs in the CTX modeling ovaries was observed by imaging under fluorescence microscopy

To examine the homing effect of hUSC transplantation via vein injection, the luciferase and GFP reporter genes in the lentivirus were constructed and transfected into hUSCs; the procedure is shown in the Fig. 2A. The cell transduction efficiency was detected by fluorescence microscopy and the results showed that more than 95% of hUSCs express GFP (Fig. 2B). In the present study, a mouse POF model was established by intraperitoneal injection of CTX with a dose of 50 mg/kg per day for 15 days. The hUSCs or hUSC-CM were transplanted into the mice by tail vein injection in three stages after 1 week of POF modeling (Fig. 2C), and the control group was only injected with hUSCs in normal mice. As shown in Fig. 2D, GFP^+^ hUSCs were significantly accumulated in the mouse ovaries of the CTX model group compared with the normal group on day 1 and day 3 after the third cell transplantation, whereas GFP^+^ hUSCs were gradually decreased on day 7 after transplantation. In addition, numerous GFP^+^ hUSCs were enriched in liver tissues in both normal and POF mice. However, a higher proportion and the absolute number of surviving GFP^+^ hUSCs were observed in the ovaries of POF mice compared with normal mice (Fig. 2D). Furthermore, our immunostaining results with the MAB1281 (red) antibody, which specifically recognizes human cells, showed that a large proportion of hUSCs still existed in the ovarian tissue within 1 week after final cell transplantation (Fig. 2E). Taken together, these results indicated that hUSCs properly homed and resided in the ovaries of mouse CTX models.

### hUSCs and hUSC-CM transplantation improved CTX-induced ovarian dysfunction through reducing the apoptosis of ovarian granulosa cells

**Fig. 3:**
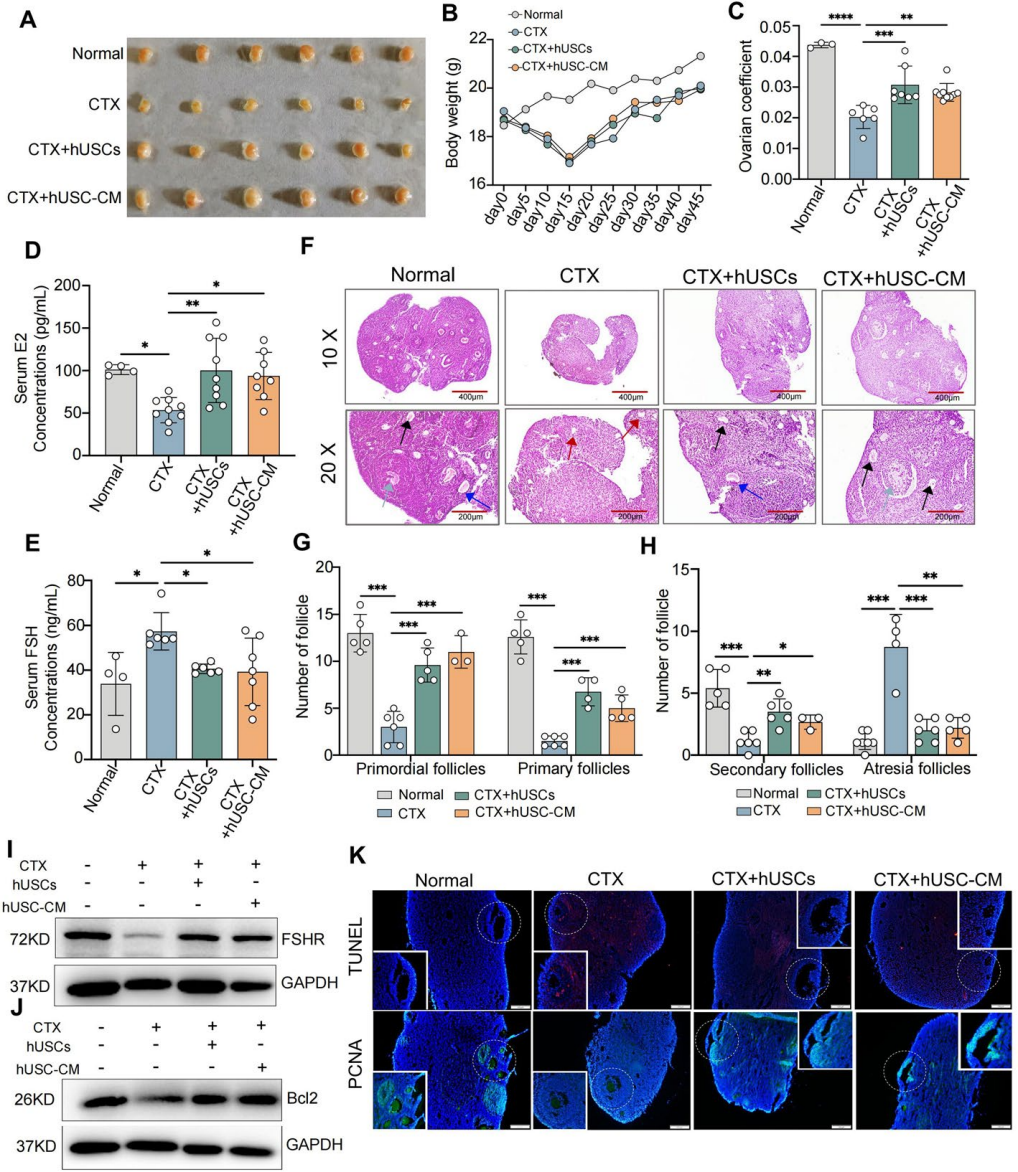
hUSCs and hUSC-CM transplantation improved CTX-induced ovarian dysfunction via inhibiting apoptosis of ovarian granulosa cells **A** Representative ovaries of each group of mice at the time of sample harvesting. **B** The weight changes of each group of mice were examined every 5 days, starting from the day before the CTX modeling. **C** The weights of mouse ovaries in different groups were measured and calculated for the ovarian coefficient. The percentage of the ovarian coefficient was calculated as: (ovarian tissue weight/body weight (g)) × 100. **D** Measurement and comparison of serum E2 between groups using ELISA. **E** Measurement and comparison of serum FSH between groups using ELISA. **F** Representative photomicrographs of H&E staining sections of mouse ovaries in the normal, CTX, CTX + hUSCs, and CTX + hUSC-CM groups. The black arrows represent primordial follicles, blue arrows represent primary follicles, gray arrows represent secondary follicles and red arrows represent atretic follicles. **G**, **H** Three non-adjacent H&E staining sections were randomly selected for counting the number of primordial follicles and primary follicles (**G**), and secondary follicles and atresia follicles (**H**) in mouse ovaries from each group. **I**, **J** Western blot assay for FHSR (**I**) and Bcl2 (**J**) expressions in ovarian tissues of different groups. **K** Detection of apoptosis and proliferation of ovarian GCs in each group using the TUNEL method and PCNA immunofluorescence staining. Significance was measured using a one-way ANOVA. *P < 0.05, **P < 0.01, ***P < 0.001

To evaluate the roles of hUSCs and hUSC-CM transplantation in CTX-induced ovarian dysfunction, the ovarian morphology was examined. The results showed that the ovaries were significantly atrophied in the CTX group compared with the normal group, while hUSCs and hUSC-CM remarkably attenuated CTX-induced atrophy of the ovaries compared with the CTX group (Fig. 3A). In addition, we observed that the mouse body weights were significantly decreased after CTX treatment and then gradually recovered (Fig. 3B). hUSCs and hUSC-CM markedly increased the ratio of ovarian weight to body weight in the CTX model mice, although they did not recover the loss of body weight in the mice during the experimental period (Fig. 3C).

Moreover, both hUSCs and hUSC-CM significantly restored the E_2_ levels and inhibited FSH secretion in CTX model mice (Fig. 3D, E). H&E staining showed that there was atrophy and a collapse of follicles in ovaries, abnormalities in the unclear follicular structure, and a reduction in the follicular numbers in the CTX group compared with the normal group; however, hUSCs and hUSC-CM significantly reversed these alternations (Fig. 3F). In addition, the results also showed that treatment with hUSCs or hUSC-CM remarkably enhanced the numbers of primordial, primary, and secondary follicles, reduced the numbers of atretic follicles (Fig. 3G, H), and enhanced the expressions of the FSH receptor (FSHR) (Fig. 3I) and Bcl2 (Fig. 3J), an anti-apoptotic protein, compared with the CTX group. Furthermore, hUSCs and hUSC-CM remarkably inhibited apoptosis and promoted the proliferation of ovarian granulosa cells (GCs) in mouse CTX models (Fig. 3K). All these results indicated that hUSCs and hUSC-CM alleviated CTX-induced ovarian dysfunctions by inhibiting the apoptosis of ovarian GCs.

### hUSC-derived exosomes (hUSC-Exo) inhibited apoptosis and enhanced the proliferation of ovarian granulosa cells in CTX-induced cellular injury in vitro

**Fig. 4:**
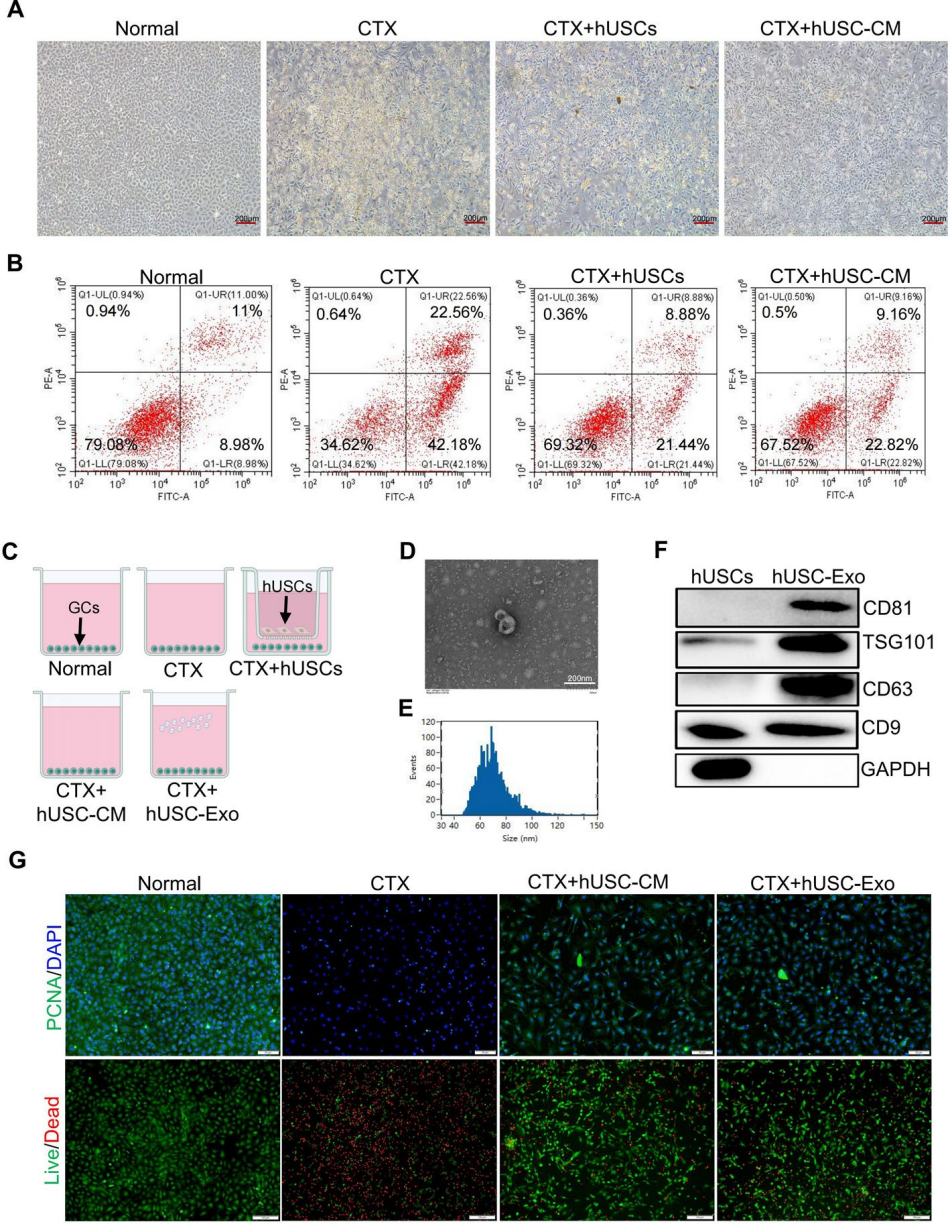
hUSCs inhibited the apoptosis and promoted the proliferation of ovarian granulosa cells induced by CTX through paracrine effects. **A**, **B** Microscopic observation of the apoptosis of GCs (**A**) and the apoptotic ratios of GCs (**B**) in the normal group, CTX group, CTX + hUSCs group, and CTX + hUSC-CM group were measured by microscope and flow cytometry. **C** In vitro GC injury models were established with CTX (5 mg/mL, 24 h) in GCs. Then, the GCs were treated with hUSCs, hUSC-CM, and hUSC-Exo for an additional 24 h. **D** A representative image of hUSC-Exo was taken under the electron microscope. **E** The particle size of hUSC-Exo was measured by particle size analysis. **F** The expressions of CD81, TSG101, CD63, and CD9 were determined by Western blot in hUSC-Exo and hUSCs. **G** Live–dead staining of cells was performed with immunofluorescence analysis to identify death and apoptosis of GCs and the expression of PCNA

To further access the roles of hUSC and hUSC-CM in CTX-induced POF in vivo, ovarian granulosa cells (GCs) were isolated to evaluate their protective effects in CTX-induced GC injury in vitro. Mouse ovarian GCs revealed a normal morphology (Supplementary Fig. 1A–C), and the immunofluorescence analysis further confirmed that GCs specifically expressed FSH receptors (Supplementary Fig. 1D). As illustrated in Fig. 4A, CTX significantly induced apoptosis and cell lysis in GCs. However, the introduction of hUSCs and hUSC-CM mitigated this apoptotic response. Quantitative analysis of the apoptosis ratio by flow cytometry revealed that CTX elevated the apoptosis rate of GCs by 40%. In contrast, both hUSCs and hUSC-CM were able to decrease the cell death rate by approximately 30% (Fig. 4B and Supplementary Fig. 2A). Next, we further determined the effects of hUSC-derived exosomes (hUSC-Exo) on GCs, since the conditioned medium contained a large number of exosomes. As shown in Fig. 4C–F, the extracted exosomes exhibited a complete structure with a particle size distribution ranging from 40 to 150 nm and high expressions of classic exosome proteins, including CD81, TSG101, CD9, and CD63. Furthermore, our PCNA immunofluorescence and live–dead staining results showed that both hUSC-CM and hUSC-Exo comparably promoted the proliferation and inhibited the apoptosis of ovarian GCs in CTX-induced models of cellular injury (Fig. 4G). These results indicated that hUSC-Exo was responsible for the protective effects of hUSCs or hUSC-CM on CTX-induced GCs injury in vitro.

### hUSC-Exo-derived miRNA-27b-3p inhibited CTX-induced apoptosis of ovarian GCs

**Fig. 5:**
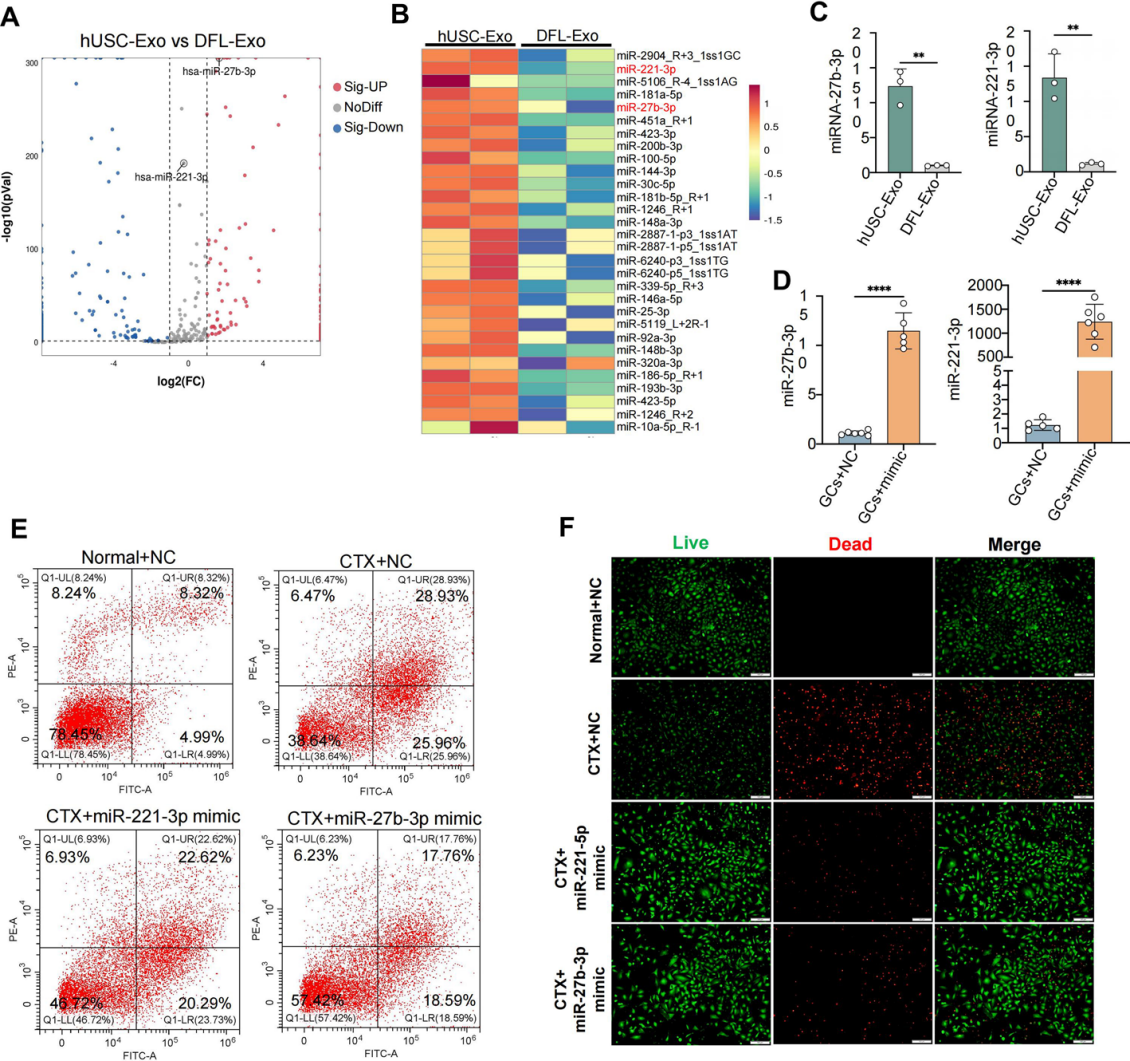
hUSC inhibited CTX-induced GC apoptosis through miRNA-221-3p and miR-27b-3p in hUSC-Exo. **A** Volcano plot of differential expression miRNAs in the hUSC-Exo and DFL-Exo groups, *n* = 3. **B** Heatmap of the differentially expressed miRNAs between hUSC-Exo and DFL-Exo (red: increased expression; blue: decreased expression). **C** High expression of miR-27b-3p and miR-221-3p in hUSC-Exo was confirmed using qPCR. **D** miR-221-3p and miR-27b-3p expression of GCs was elevated after transient overexpression of miR-221-3p and miR-27b-3p. **E** The apoptotic ratios of GCs in the normal + NC group, CTX + NC group, CTX + miR-221-3p mimic group, and CTX + miR-27b-3p mimic group were detected by flow cytometry. **F** Live–dead staining to identify apoptosis of GCs was conducted using immunofluorescence staining *P < 0.05, **P < 0.01, ***P < 0.001, ****P < 0.0001

The miRNA profiles of exosomes derived from hUSCs were determined using miRNA-sequencing to understand their role in CTX-induced GC injury. We compared and analyzed the expressions of miRNAs in hUSC-Exo and DFL-Exo by generating a volcano plot to illustrate our findings. The results demonstrated that a substantial number of miRNAs, including miR-27b-3p, were highly expressed in hUSC-Exo while exhibiting low expression levels in the DFL-Exo (exosomes derived from the most commonly used negative control cells in MSC exosome miRNA sequencing, Fig. 5A). We screened 30 miRNAs that were highly expressed in hUSC-Exo and 30 miRNAs that were highly expressed in DFL-Exo to create a heat map. The analysis results showed that there were significant differences between miR-27b-3p and miR-221-3p. Overall, miR-27b-3p exhibited the most pronounced difference (Fig. 5B). The gene ontology (GO) analysis (https://geneontology.org/) revealed that the downstream genes targeted by differentially expressed miRNAs were predominantly enriched in processes related to multicellular organism development and protein binding. Multicellular organism development was closely associated with the development of ovarian germ cells, while protein binding played a crucial role in the regulation of cell apoptosis (Supplementary Fig. 3A). The top 20 biological process enrichment analyses revealed that multicellular organism development was highly significant. GO analysis results suggested that miRNA in hUSC-Exo primarily targeted genes involved in regulating ovarian development and cell apoptosis (Supplementary Fig. 3B). The Kyoto Encyclopedia of Genes and Genomes (KEGG) enrichment analysis (https://www.kegg.jp/) revealed that the PI3K-AKT signaling pathway had the highest number of enriched downstream target genes from differentially expressed miRNAs (Supplementary Fig. 4A). Furthermore, the top 20 enrichment analyses from the KEGG results indicated that the PI3K-Akt signaling pathway ranked second in terms of significance of enrichment (Supplementary Fig. 4B). The qPCR results showed that the expressions of miRNA-27b-3p was much more than that of miR-221-3p in the hUSC-Exo compared with the DFL-Exo (Fig. 5C). Moreover, following transfection of GCs with a constructed miRNA mimic, qPCR analysis revealed that the expression of miRNA-27b-3p was also higher than that of miRNA-221-3p in GCs (Fig. 5D). Flow cytometry results demonstrated that although the overexpression of both miRNA-27b-3p and miRNA-221-3p effectively inhibited CTX-induced apoptosis of GCs, miRNA-27b-3p exhibited a superior effect compared with miRNA-221-3p (Fig. 5E and Supplementary Fig. 2B). In addition, live–death staining results further supported that the inhibitory effect of miRNA-27b-3p on CTX-induced death of GCs was also better than that of miRNA-221-3p (Fig. 5F). Taken together, our results suggest that hUSC-Exo might regulate the downstream target genes which are involved in inhibiting cell apoptosis and promoting cell development in the ovary via miRNA-27b-3p.

### RNA-sequencing uncovered the target genes and signaling pathways of hUSC-Exo in inhibiting CTX-induced POF

**Fig. 6:**
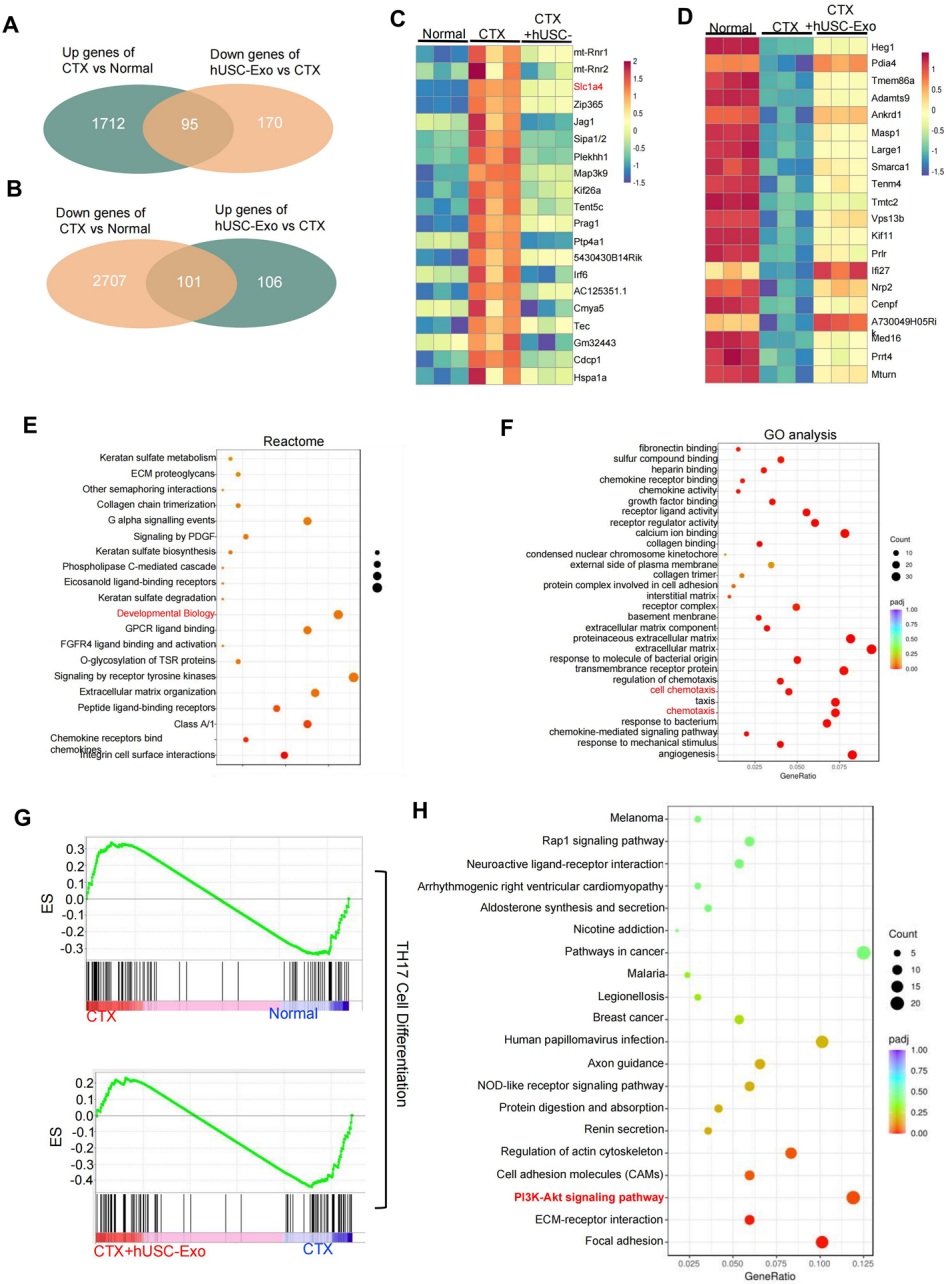
The related genes and signal pathways that have an antiapoptotic role associated with hUSC-Exo were determined in CTX-induced GC-injury models. **A**, **B** Venn diagram statistics for up- or downregulated differential genes between the CTX group, CTX + hUSC-Exo group, and CTX + hUSC-Exo group, respectively. **C**, **D** Heat map of differential genes was obtained from Venn plot statistics using differential expression ploidy. **E** Reactome analysis of the differential genes. **F** GO enrichment analysis of the differential genes. **G** The expressions of genes causing Th17 cell differentiation were determined using GSEA analysis in the different groups. **H** KEGG analysis of the differential genes revealed that most of them were enriched in the AKT signaling pathway

To further investigate the mechanism by which hUSC-Exo alleviated the apoptosis of GCs, transcriptome sequencing was performed in GCs treated with CTX and CTX + hUSC-Exo. The principal component analysis revealed a distinct difference in three groups, without major deviations for the three replicate samples within each group (Supplementary Fig. 5A). In addition, the transcriptome sequencing results showed that 1808 genes were upregulated and 2808 genes were downregulated in the CTX group compared with the normal group (Supplementary Fig. 5B), and 207 genes were upregulated and 265 genes were downregulated in the hUSC-Exo group compared with the CTX group (Supplementary Fig. 5C). Moreover, the Venn analysis showed that 95 genes were overlapped between the upregulated genes in the CTX group and the downregulated genes in the hUSC-Exo group (Fig. 6A), and 101 genes were overlapped between the downregulated genes in the CTX group and the upregulated genes in the hUSC-Exo group (Fig. 6B). In addiiton, a heat map analysis revealed that there were significant differences in the expressions of Heg1, Pdia4, Tmem86a, Ankrd1, and Slc1a4 genes among the three groups (Supplementary Table 3–4, Fig. 6C, D). Reactome analysis (https://reactome.org/) showed that differential genes were enriched in biological development-related pathways, which was also consistent with the GO analysis (https://geneontology.org/) results of miRNA sequencing (Fig. 6E). The results of the GO analysis showed that differential genes were related to the chemokines and cell chemotaxis. Chemokines can enhance the antiapoptotic activity of cells through the PI3K pathway (Fig. 6F). Besides, the GSEA analysis showed that TH17 cell differentiation was significantly upregulated by CTX treatment, whereas hUSC-Exo remarkably inhibited CTX-induced TH17 cell differentiation, indicating that hUSC-Exo reduced the CTX-induced inflammatory response in GCs (Fig. 6G). The KEGG enrichment analysis (https://www.kegg.jp/) showed that most of the differential genes were also related to the PI3K-Akt signaling pathway that regulates cell proliferation and apoptosis (Fig. 6H). The above results indicated that the target genes of hUSC-Exo had the function of regulating GC apoptosis and normal ovarian development through the PI3K-AKT pathway.

### Knockdown of SLC1A4 inhibited CTX-induced apoptosis of GCs by inhibiting the outflux of intracellular serine

**Fig. 7:**
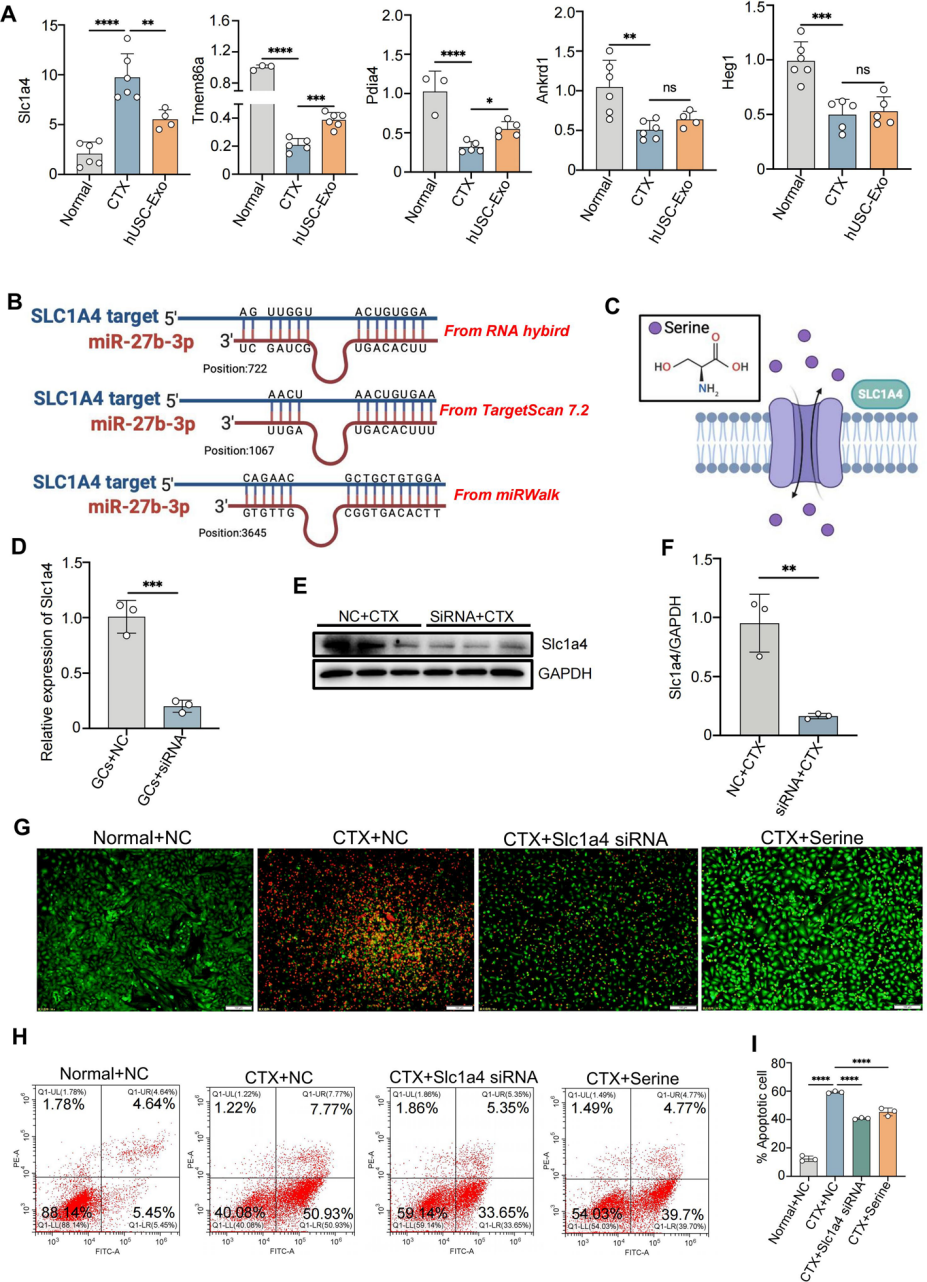
miR-27b-3p inhibited the outflux of intracellular serine by selectively suppressing the expressions of SLC1A4 in CTX-induced POF. **A** The expressions of Slc1a4, Tmem86a, Pdia4, Ankrd1, and Heg1 were detected in GCs by qRT-PCR. **B** Predicted target sites of *Slc1a4* 3′UTR (red letters show the predicted pairing of the target region and miRNA). **C** Schematic diagram of SLC1A4 and serine transmembrane transport. **D**, **E** Detection of knockdown efficiency of Slc1a4 on GCs after siRNA transfection by qPCR and Western blotting. **F** The relative intensity of Slc1a4 in Western blotting. **G** Live–dead staining was used to detect death in the normal + NC group, CTX + NC group, CTX + siRNA group, and CTX + serine group. **H** The apoptotic ratios of GCs in each group were detected by flow cytometry. **I** The apoptosis of GCs was quantitatively detected by flow cytometry *P < 0.05, **P < 0.01, ***P < 0.001, ****P < 0.0001

To verify the sequencing results, the expressions of the five differential genes were examined with qPCR. The results showed that the expression of SLC1A4 was significantly upregulated in the CTX group and remarkably downregulated in the hUSC-Exo group, respectively (Fig. 7A), and that the expressions of other genes, including *Tmem86a*, *Pdia4*, *Ankrd1* and *Heg1* were inhibited by CTX or hUSC-Exo. In addition, through the prediction of RNA hybridization, TargetScan7.2 (http://www.targetscan.org/vert_72/), and miRWalk databases (http://mirwalk.umm.uni-heidelberg.de) we identified that there were three binding sites in the SLC1A4 locus for miR-27b-3p, suggesting that SLC1A4 might be the target gene of miR-27b-3p (Fig. 7B). To further elucidate the role of SLC1A4 in POF, SLC1A4 was knocked down in GCs via siRNA and confirmed by qPCR and Western blotting. The results showed the expression of SLC1A4 was significantly decreased after siRNA transfection (Fig. 7C–E). Furthermore, our immunofluorescent staining results showed that the numbers of dead GCs were significantly decreased in the SLC1A4 knockdown or serine supplementation groups compared with the CTX group (Fig. 7F). Moreover, the flow cytometry results also showed that SLC1A4 knockdown or serine supplementation remarkably inhibited the apoptosis of ovarian GCs (Fig. 7G–H). All these results indicated that SLC1A4 knockdown alleviated CTX-induced GC apoptosis by suppressing the outflux of the intracellular serine.

### hUSC-Exo inhibited CTX-induced GC apoptosis by activating the AKT/mTOR signaling pathway

**Fig. 8:**
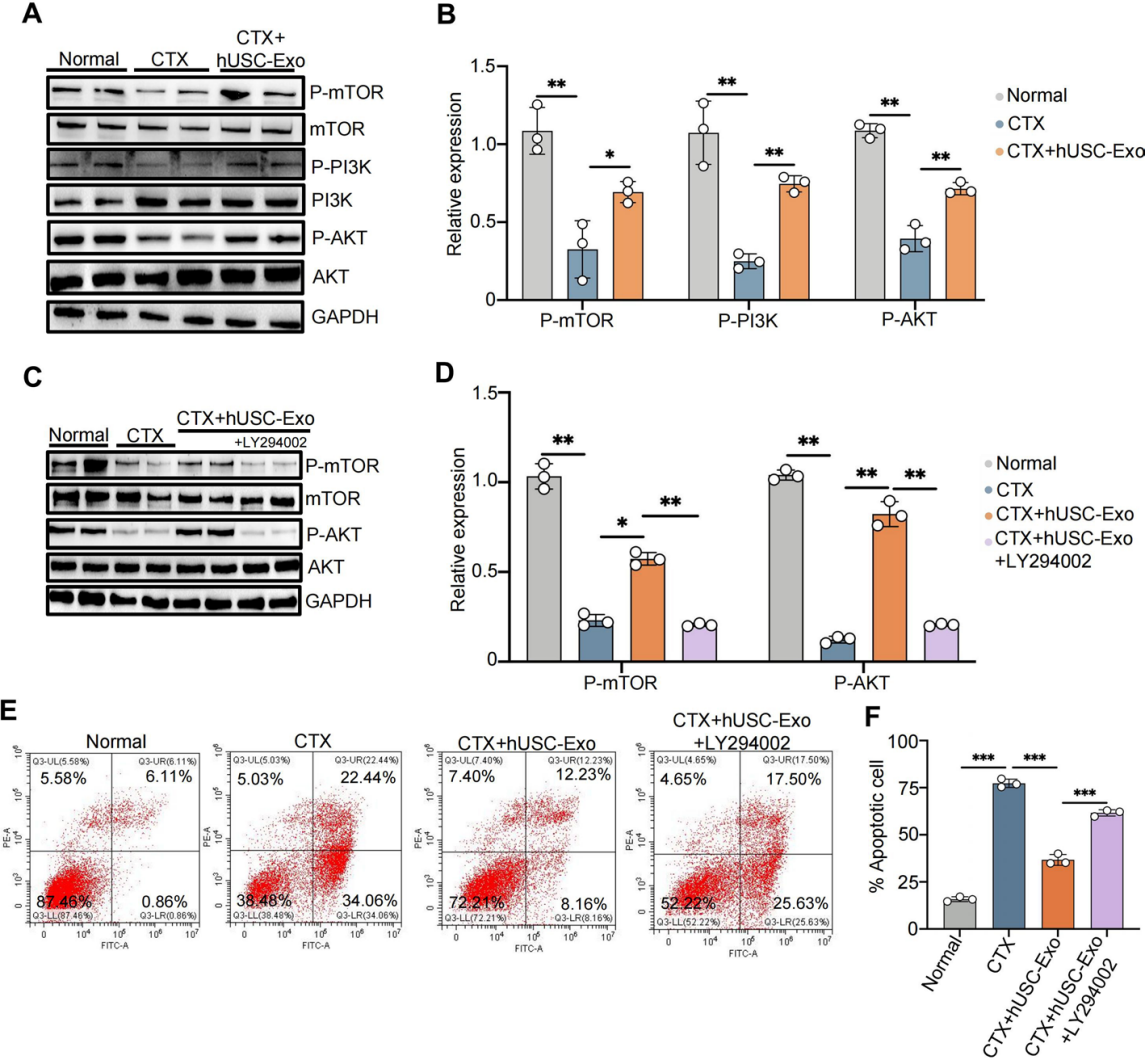
hUSC-Exo inhibited CTX-induced apoptosis of GCs by activating the PI3K/AKT/mTOR signal pathway. **A**, **B** The expressions of the proteins related to the PI3K/Akt/mTOR signaling pathway, such as P-mTOR, P-PI3K ,and P-AKT, were detected by Western blot analysis in GCs in the treatment of hUSC-Exo. **C**, **D** The expressions of the proteins related to the PI3K/Akt/mTOR signaling pathway were determined by Western blot analysis in GCs in the treatment of hUSC-Exo with or without LY294002, an inhibitor of the PI3K/AKT/mTOR signaling pathway. **E** The apoptotic ratios of GCs in the normal group, CTX group, CTX + hUSC-Exo group, and CTX + hUSC-Exo + LY294002 group were detected by flow cytometry. **F** The quantitative results were obtained from the flow cytometry (**E**) *P < 0.05, **P < 0.01

It has been demonstrated that serine could bind to palmitoyl-CoA to form sphingosine in ovaries, which regulates the proliferation and apoptosis of GCs through the PI3K/AKT/mTOR signaling pathway [21]. Our sequencing results showed that the activation of the PI3K/AKT signaling pathway was associated with the protective roles of hUSC-Exo in the CTX group. To further clarify the protective mechanisms of hUSC-Exo in CTX-induced GC apoptosis, we examined the expressions of the genes involved in PI3K/AKT signaling. The results showed that the phosphorylation of PI3K, AKT, and mTOR was significantly downregulated in the CTX group, while hUSC-Exo remarkably elevated the phosphorylation of these genes (Fig. 8A, B). Furthermore, the elevated phosphorylation of PI3K, AKT, and mTOR was markedly reversed by LY294002, an PI3K/AKT signaling inhibitor (Fig. 8C, D), and the flow cytometry results also showed that the inhibitory effect of hUSC-Exo on CTX-induced GC apoptosis was blocked by LY294002 (Fig. 8E, F). Taken together, these results indicated that hUSC-Exo inhibited CTX-induced GC apoptosis through the activation of the AKT/mTOR signaling pathway.

## Discussion

Cyclophosphamide (CTX) is one of the most common chemotherapeutic drugs that often leads to POF in young women [22]. However, the underlying mechanism is not clear. Existing studies suggest that CTX-induced POF might be associated with the elevated apoptosis of GCs [23] or oocytes [24]. Recent studies have reported that MSCs derived from bone marrow, adipose, the umbilical cord, and other tissues were able to alleviate the symptoms of CTX-induced POF in mice [9, 25–27], and that MSCs exerted therapeutic effects through the paracrine mechanism rather than through differentiation into granular cells [28–30]. In the present study, we showed that human urine-derived stem cells (hUSCs) are easily obtained from urine and possess the typical characteristics of stem cells, such as the potent abilities of self-renewal, proliferation, and multidirectional differentiation. Besides the features described above, our study also identified that hUSCs had the advantages of low immunogenicity and no tumorigenicity. Studies observed that hUSCs had therapeutic potential in diseases such as liver fibrosis, acute liver injury, diabetes, cartilage defects, skin damage, and acute lung injury [31]. The results from others and our studies showed that there was no significant difference between male and female derived hUSCs [32, 33]. Here, we demonstrated that hUSCs and hUSC-CM significantly improved CTX-induced dysfunctions of ovaries equally, as evidenced by the restoration of the atrophied ovaries, elevation of E2 levels, and suppression of FSH secretion, suggesting that the protective effect was related to their paracrine signaling. It has been reported that ovarian granulosa cells (GCs) promoted oocyte development and secretion of estrogen [34]. Our results from Tunel and PCNA staining showed that hUSCs remarkably inhibited CTX-induced apoptosis and promoted the proliferation of ovarian GCs, indicating that hUSCs protected mice from CTX-induced POF, mainly through inhibiting the apoptosis of GCs.

Although our results showed that the hUSCs preferably reside in the damaged ovaries induced by CTX, the protective effect of hUSCs on POF was mainly related to their paracrine signaling, since both hUSCs and hUSC-CM had an equal protective effect. MSC-derived conditioned medium contains cytokines, exosomes etc. [35]. To determine the key substances of hUSC-CM that inhibited the apoptosis of GCs, hUSC-derived exosomes (hUSC-Exo) were prepared from hUSC-CM, and the effects of both hUSC-Exo and hUSC-CM on the CTX-induced injury model of GCs were evaluated in vitro. Our results indicate that both hUSCs-CM and hUSC-Exo significantly reduced CTX-induced apoptosis of GCs equally, suggesting that hUSC-Exo might contain the key elements for protecting the GCs from CTX-induced injury.

Exosomes serve as an intercellular communicator, in which exosome-derived miRNA plays a pivotal role in many biological processes. It has been reported that exosomal miR-21 derived from BM-MSCs inhibited the apoptosis of GCs by suppressing the expressions of PDCD4 and PTEN in GCs [28]. In addition, miR-644-5p [29] and miR-144-5p [30] in exosomes from BM-MSCs also played important roles in alleviating POF. Moreover, miR-320a in exosomes of human amniotic mesenchymal stem cells (hAMSCs) inhibited POF via the regulation of target genes SIRT4. Furthermore, miR-10a in exosomes of amniotic fluid stem cells (AFSCs) also improved POF by inhibiting apoptosis of GCs [36, 37]. However, the roles of miRNA in hUSC-Exo in CTX-induced POF have not been reported. In our study, we identified that miR-221-3p and miR-27b-3p were significantly upregulated in hUSC-Exo compared with DFL-Exo. It has been reported that miR-27b-3p inhibits microglia inflammatory responses and cell apoptosis [38]. Our results showed that overexpression of miR-27b-3p significantly inhibited CTX-induced apoptosis of GCs, suggesting that exosomal miR-27b-3p derived from hUSCs might contribute to the protective effect of hUSCs on CTX-induced POF.

SLC1A4 (also referred to as ASCT1), a member of the SLC family, is a neutral amino acid transporter that transports neutral amino acids, including cysteine, serine, and alanine [16]. It has been reported that SLC1A4 plays an important role in diseases related to central nervous system injury by regulating serine transport; SLC1A4 was upregulated in mice with central nerve injury, and SLC1A4 deficiency protected the injury [39]. In addition, it has been observed that SLC1A4 protein expression in isolated brain microvessels and brain prefrontal cortices in 5xFAD mice (Alzheimer’s disease) was twice as high as that in wild-type (WT) controls [40]. Our sequencing results showed that SLC1A4 was substantially upregulated in the CTX group, whereas hUSCs and hUSC-Exo significantly inhibited the expression of SLC1A4 in GCs. It has been reported that the content of serine in the ovaries of the POF model was dramatically decreased [21]. Serine is an important molecule for sphingolipid synthesis, in which sphingolipid participates in the activation of the PI3K/Akt/mTOR signaling pathway and is also essential for ovarian development, follicle growth, and proliferation and differentiation of GCs [21]. Our results showed that hUSC-Exo protected GCs from CTX-induced apoptosis through selectively activating the PI3K/Akt signaling pathway. Importantly, according to the prediction, miR-27b-3p could bind to the mRNA of the SLC1A4 gene and thus inhibit the expression of SLC1A4. In the present study, we observed that SLC1A4 knockdown significantly reduced CTX-induced GC apoptosis. Meanwhile, serine supplementation also remarkably inhibited CTX-induced GC apoptosis. These results indicate that the elevated expression of SLC1A4 promotes the translocation of the intracellular serine to the extracellular compartment, resulting in a decrease in the intracellular serine content under CTX treatment. In contrast, *Slc1a4* knockdown inhibited the outflow of serine from the cells, in which the elevated intracellular serine content was favorable to the synthesis of sphingolipids, leading to the activation of the PI3K/Akt/mTOR signaling pathway owing to the phosphorylation of sphingolipids. Therefore, insufficient sphingolipid synthesis will lead to the inability of the PI3K/Akt/mTOR signaling pathway to be activated effectively, leading to the apoptosis of GCs and ovarian atrophy. Our results demonstrated that the miR-27b-3p in hUSC-Exo could bind to SLC1A4 in GCs to inhibit the protein expression of SLC1A4, thereby reducing the intracellular serine transport to the extracellular compartment and maintaining the intracellular serine content. Moreover, our results showed that the PI3K/Akt/mTOR signaling pathway was activated in the hUSC-Exo group, and the inhibition of GC apoptosis by hUSC-Exo was also blocked by the PI3K/Akt/mTOR signaling pathway inhibitor LY294002, indicating that the protective effect of hUSCs on CTX-induced apoptosis of GCs was related to activation of the PI3K/Akt/mTOR signaling pathway.

However, there are still some limitations in this study. First, the specific molecular mechanisms underlying CTX-induced premature ovarian failure should be further investigated. Second, serine supplementation elevates the levels of female hormones, which may reduce the therapeutic effect of CTX since some inhibitor of female hormone is applied the patients with breast. Third, the medical compliance problem of serine application should be addressed since the administration of serine usually lasts for several months. Given these limitations, in the future, we will further investigate the effects of SLC1A4 and serine metabolism on POF. Specifically, we will explore whether SLC1A4 is the most critical protein in chemotherapeutic drug-induced POF using SLC1A4 conditioned knockout mice. At the same time, we will also investigate whether simple serine supplementation will be able to prevent or inhibit CTX-induced POF.

In summary, we demonstrated that CTX-induced apoptosis of ovarian GCs was essential for POF, in which CTX reduced the intracellular serine level by increasing the expression of SLC1A4 in ovarian GCs, and in turn, promoted the apoptosis of GCs through the inhibition of the PI3K/Akt/mTOR signaling pathway, which, eventually, induced POF. The underlying mechanism of the protective effect of hUSCs on CTX-induced POF was related to hUSC-derived exosomal miR-27b-3p, which inhibited GC apoptosis and promoted ovarian growth by specifically targeting SLC1A4 in ovarian GCs. In turn, the elevated serine improved CTX-induced POF by activating the PI3K/AKT/mTOR signaling pathway. Certainly, our study suggests that hUSC-based cell therapy or simple supplementation of serine may be an efficient therapeutic approach for the prevention and treatment of POF clinically.

## Conclusions

The results of the present study suggest that hUSC and hUSC-CM significantly alleviated CTX-induced ovarian damages and GC apoptosis. Exosomal miR-27b-3p derived from hUSCs reduced CTX-induced GC apoptosis by regulating Slc1a4 and then maintaining the balance of intracellular and extracellular serine levels, thus activating the PI3K/Akt/mTOR signaling pathway to promote the proliferation of GCs.

## Supplementary Information

Supplementary Material 1.

## Data Availability

The data that support the findings of this study are available from the corresponding author upon reasonable request.

## Abbreviations

CTX: Cyclophosphamide
POF: Premature ovarian failure
hUSCs: Human urine stem cells
GCs: Ovarian granulosa cells
FSH: Follicle-stimulating hormone
MSCs: Mesenchymal stem cells
E2: Estrogen
hUSC-Exo: HUSC-derived exosomes
hAMSCs: Human amniotic mesenchymal stem cells
SLC1A4: Solute carrier family 1, member 4

## References

[1] van Hellemond IEG, Vriens IJH, Peer PGM, Swinkels ACP, Smorenburg CH, Seynaeve CM, et al. Ovarian function recovery during anastrozole in breast cancer patients with chemotherapy-induced ovarian function failure. J Natl Cancer Inst. 2017. 10.1093/jnci/djx074. 29546343 10.1093/jnci/djx074

[2] Torrealday S, Kodaman P, Pal L. Premature ovarian insufficiency–an update on recent advances in understanding and management. F1000Res. 2017;6:2069. 29225794 10.12688/f1000research.11948.1 PMC5710309

[3] Liu T, Li Q, Wang S, Chen C, Zheng J. Transplantation of ovarian granulosa-like cells derived from human induced pluripotent stem cells for the treatment of murine premature ovarian failure. Mol Med Rep. 2016;13(6):5053–8. 27121006 10.3892/mmr.2016.5191 PMC4878559

[4] Song D, Zhong Y, Qian C, Zou Q, Ou J, Shi Y, et al. Human umbilical cord mesenchymal stem cells therapy in cyclophosphamide-induced premature ovarian failure rat model. Biomed Res Int. 2016;2016:2517514. 27047962 10.1155/2016/2517514 PMC4800076

[5] Esfandyari S, Chugh RM, Park HS, Hobeika E, Ulin M, Al-Hendy A. Mesenchymal stem cells as a bio organ for treatment of female infertility. Cells. 2020;9(10):2253. 33050021 10.3390/cells9102253 PMC7599919

[6] Ghahremani-Nasab M, Ghanbari E, Jahanbani Y, Mehdizadeh A, Yousefi M. Premature ovarian failure and tissue engineering. J Cell Physiol. 2020;235(5):4217–26. 31663142 10.1002/jcp.29376

[7] Liu T, Huang Y, Zhang J, Qin W, Chi H, Chen J, et al. Transplantation of human menstrual blood stem cells to treat premature ovarian failure in mouse model. Stem Cells Dev. 2014;23(13):1548–57. 24593672 10.1089/scd.2013.0371 PMC4066227

[8] Wang S, Yu L, Sun M, Mu S, Wang C, Wang D, et al. The therapeutic potential of umbilical cord mesenchymal stem cells in mice premature ovarian failure. Biomed Res Int. 2013;2013: 690491. 23998127 10.1155/2013/690491 PMC3753743

[9] He Y, Chen D, Yang L, Hou Q, Ma H, Xu X. The therapeutic potential of bone marrow mesenchymal stem cells in premature ovarian failure. Stem Cell Res Ther. 2018;9(1):263. 30286808 10.1186/s13287-018-1008-9 PMC6172726

[10] Chen AJ, Pi JK, Hu JG, Huang YZ, Gao HW, Li SF, et al. Identification and characterization of two morphologically distinct stem cell subpopulations from human urine samples. Sci China Life Sci. 2020;63(5):712–23. 31515730 10.1007/s11427-018-9543-1

[11] Song YT, Li YQ, Tian MX, Hu JG, Zhang XR, Liu PC, et al. Application of antibody-conjugated small intestine submucosa to capture urine-derived stem cells for bladder repair in a rabbit model. Bioact Mater. 2022;14:443–55. 35415280 10.1016/j.bioactmat.2021.11.017 PMC8978277

[12] Galhom RA, Korayem HE, Ibrahim MA, Abd-Eltawab Tammam A, Khalifa MM, Rashwan EK, et al. Urine-derived stem cells versus their lysate in ameliorating erectile dysfunction in a rat model of type 2 diabetes. Front Physiol. 2022;13: 854949. 35620604 10.3389/fphys.2022.854949 PMC9127444

[13] Jiang ZZ, Liu YM, Niu X, Yin JY, Hu B, Guo SC, et al. Exosomes secreted by human urine-derived stem cells could prevent kidney complications from type I diabetes in rats. Stem Cell Res Ther. 2016;7:24. 26852014 10.1186/s13287-016-0287-2 PMC4744390

[14] Zhang W, Hu J, Huang Y, Wu C, Xie H. Urine-derived stem cells: applications in skin, bone and articular cartilage repair. Burns Trauma. 2021;9:tkab039. 34859109 10.1093/burnst/tkab039 PMC8633594

[15] Yang B, Wang J, Qiao J, Zhang Q, Liu Q, Tan Y, et al. Circ DENND4C inhibits pyroptosis and alleviates ischemia-reperfusion acute kidney injury by exosomes secreted from human urine-derived stem cells. Chem Biol Interact. 2024;391: 110922. 38412628 10.1016/j.cbi.2024.110922

[16] Peng X, Chen R, Cai S, Lu S, Zhang Y. SLC1A4: a powerful prognostic marker and promising therapeutic target for HCC. Front Oncol. 2021;11: 650355. 33777811 10.3389/fonc.2021.650355 PMC7991385

[17] Handzlik MK, Metallo CM. Sources and sinks of serine in nutrition, health, and disease. Annu Rev Nutr. 2023;43:123–51. 37307855 10.1146/annurev-nutr-061021-022648 PMC10783795

[18] Kaplan E, Zubedat S, Radzishevsky I, Valenta AC, Rechnitz O, Sason H, et al. ASCT1 (Slc1a4) transporter is a physiologic regulator of brain d-serine and neurodevelopment. Proc Natl Acad Sci U S A. 2018;115(38):9628–33. 30185558 10.1073/pnas.1722677115 PMC6156681

[19] Zhou T, Benda C, Dunzinger S, Huang Y, Ho JC, Yang J, et al. Generation of human induced pluripotent stem cells from urine samples. Nat Protoc. 2012;7(12):2080–9. 23138349 10.1038/nprot.2012.115

[20] Guo F, Xia T, Zhang Y, Ma X, Yan Z, Hao S, et al. Menstrual blood derived mesenchymal stem cells combined with Bushen Tiaochong recipe improved chemotherapy-induced premature ovarian failure in mice by inhibiting GADD45b expression in the cell cycle pathway. Reprod Biol Endocrinol. 2019;17(1):56. 31311554 10.1186/s12958-019-0499-2 PMC6636150

[21] Zhao Y, Ma J, Yi P, Wu J, Zhao F, Tu W, et al. Human umbilical cord mesenchymal stem cells restore the ovarian metabolome and rescue premature ovarian insufficiency in mice. Stem Cell Res Ther. 2020;11(1):466. 33148334 10.1186/s13287-020-01972-5 PMC7641864

[22] Haukvik UK, Dieset I, Bjoro T, Holte H, Fossa SD. Treatment-related premature ovarian failure as a long-term complication after Hodgkin’s lymphoma. Ann Oncol. 2006;17(9):1428–33. 16831852 10.1093/annonc/mdl149

[23] Lin L, Gao W, Chen Y, Li T, Sha C, Chen L, et al. Reactive oxygen species-induced SIAH1 promotes granulosa cells’ senescence in premature ovarian failure. J Cell Mol Med. 2022;26(8):2417–27. 35261172 10.1111/jcmm.17264 PMC8995443

[24] Lee HJ, Selesniemi K, Niikura Y, Niikura T, Klein R, Dombkowski DM, et al. Bone marrow transplantation generates immature oocytes and rescues long-term fertility in a preclinical mouse model of chemotherapy-induced premature ovarian failure. J Clin Oncol. 2007;25(22):3198–204. 17664466 10.1200/JCO.2006.10.3028

[25] Zheng Q, Fu X, Jiang J, Zhang N, Zou L, Wang W, et al. Umbilical cord mesenchymal stem cell transplantation prevents chemotherapy-induced ovarian failure via the NGF/TrkA pathway in rats. Biomed Res Int. 2019;2019:6539294. 31240219 10.1155/2019/6539294 PMC6556346

[26] Ding L, Yan G, Wang B, Xu L, Gu Y, Ru T, et al. Transplantation of UC-MSCs on collagen scaffold activates follicles in dormant ovaries of POF patients with long history of infertility. Sci China Life Sci. 2018;61(12):1554–65. 29546669 10.1007/s11427-017-9272-2

[27] Ling L, Feng X, Wei T, Wang Y, Wang Y, Wang Z, et al. Human amnion-derived mesenchymal stem cell (hAD-MSC) transplantation improves ovarian function in rats with premature ovarian insufficiency (POI) at least partly through a paracrine mechanism. Stem Cell Res Ther. 2019;10(1):46. 30683144 10.1186/s13287-019-1136-x PMC6347748

[28] Fu X, He Y, Wang X, Peng D, Chen X, Li X, et al. Overexpression of miR-21 in stem cells improves ovarian structure and function in rats with chemotherapy-induced ovarian damage by targeting PDCD4 and PTEN to inhibit granulosa cell apoptosis. Stem Cell Res Ther. 2017;8(1):187. 28807003 10.1186/s13287-017-0641-z PMC5556338

[29] Sun B, Ma Y, Wang F, Hu L, Sun Y. miR-644-5p carried by bone mesenchymal stem cell-derived exosomes targets regulation of p53 to inhibit ovarian granulosa cell apoptosis. Stem Cell Res Ther. 2019;10(1):360. 31783913 10.1186/s13287-019-1442-3 PMC6884862

[30] Yang M, Lin L, Sha C, Li T, Zhao D, Wei H, et al. Bone marrow mesenchymal stem cell-derived exosomal miR-144–5p improves rat ovarian function after chemotherapy-induced ovarian failure by targeting PTEN. Lab Invest. 2019. 10.1038/s41374-019-0321-y. 31537899 10.1038/s41374-019-0321-y

[31] PavathuparambilAbdulManaph N, Al-Hawwas M, Bobrovskaya L, Coates PT, Zhou XF. Urine-derived cells for human cell therapy. Stem Cell Res Ther. 2018;9(1):189. 29996911 10.1186/s13287-018-0932-z PMC6042455

[32] Boysen AT, Whitehead B, Revenfeld ALS, Gupta D, Petersen T, Nejsum P. Urine-derived stem cells serve as a robust platform for generating native or engineered extracellular vesicles. Stem Cell Res Ther. 2024;15(1):288. 39256816 10.1186/s13287-024-03903-0 PMC11389316

[33] Burdeyron P, Giraud S, Hauet T, Steichen C. Urine-derived stem/progenitor cells: a focus on their characterization and potential. World J Stem Cells. 2020;12(10):1080–96. 33178393 10.4252/wjsc.v12.i10.1080 PMC7596444

[34] Liu R, Zhang X, Fan Z, Wang Y, Yao G, Wan X, et al. Human amniotic mesenchymal stem cells improve the follicular microenvironment to recover ovarian function in premature ovarian failure mice. Stem Cell Res Ther. 2019;10(1):299. 31578152 10.1186/s13287-019-1315-9 PMC6775662

[35] Culenova M, Nicodemou A, Novakova ZV, Debreova M, Smolinska V, Bernatova S, et al. Isolation, culture and comprehensive characterization of biological properties of human urine-derived stem cells. Int J Mol Sci. 2021;22(22):12503. 34830384 10.3390/ijms222212503 PMC8624597

[36] Ding C, Qian C, Hou S, Lu J, Zou Q, Li H, et al. Exosomal miRNA-320a is released from hAMSCs and regulates SIRT4 to prevent reactive oxygen species generation in POI. Mol Ther Nucleic Acids. 2020;21:37–50. 32506013 10.1016/j.omtn.2020.05.013 PMC7272510

[37] Xiao GY, Cheng CC, Chiang YS, Cheng WT, Liu IH, Wu SC. Exosomal miR-10a derived from amniotic fluid stem cells preserves ovarian follicles after chemotherapy. Sci Rep. 2016;6:23120. 26979400 10.1038/srep23120 PMC4793229

[38] Li L, Qi C, Liu Y, Shen Y, Zhao X, Qin H, et al. MicroRNA miR-27b-3p regulate microglial inflammation response and cell apoptosis by inhibiting A20 (TNF-alpha-induced protein 3). Bioengineered. 2021;12(2):9902–13. 34895052 10.1080/21655979.2021.1969195 PMC8810141

[39] Tapanes SA, Arizanovska D, Diaz MM, Folorunso OO, Harvey T, Brown SE, et al. Inhibition of glial D-serine release rescues synaptic damage after brain injury. Glia. 2022;70(6):1133–52. 35195906 10.1002/glia.24161 PMC9305835

[40] Puris E, Saveleva L, de Sousa MI, Kanninen KM, Auriola S, Fricker G. Protein expression of amino acid transporters is altered in isolated cerebral microvessels of 5xFAD mouse model of Alzheimer’s disease. Mol Neurobiol. 2023;60(2):732–48. 36367657 10.1007/s12035-022-03111-y PMC9849299

