# Supplementary material for: Human urine stem cells protect against cyclophosphamide-induced premature ovarian failure by inhibiting SLC1A4-mediated outflux of intracellular serine in ovarian granulosa cells
Source: Cell Mol Biol Lett. 2025 Feb 19;30:21. doi: 10.1186/s11658-025-00701-1 (PMC11840982; doi:10.1186/s11658-025-00701-1)
Supplement: Supplementary file 1 — Supplementary Material 1. [file 11658_2025_701_MOESM1_ESM.docx]

**Supporting Information**

**Human urine stem cells protect against cyclophosphamide-induced premature ovarian failure via inhibiting SLC1A4-mediated the outflux of intracellular serine in ovarian granulosa cells**

Hao-Cheng Gu^1,2,#^, Ling-Fang Wang^1,#^, Yu-Wei Zhang^1,2^, You-Qiong Zhuo^1,3^, Zhou-Hang Zhang^1^, Xing-Yu Wei^1^, Quan-Wen Liu^1^, Ke-Yu Deng^1,2*^ and Hong-Bo Xin^1,2,3*^

**Supplementary Materials and Methods. Isolation, culture, and identification of ovarian GCs**

The 3-week-old female mice were injected intraperitoneally with pregnant mare serum gonadotropin (PMSG; Solarbio, China) to stimulate follicle growth. The mice were sacrificed by cervical dislocation and the mouse were dissected after 48 h of injection. The adipose tissue and connective tissue attached to the ovaries were peeled off in a pre-chilled PBS solution. The ovarian GCs were released by puncturing the follicles with a 1 mL syringe under a body vision microscope. The cells were washed with PBS for three times and centrifuged at 1000 rpm for 5 min. The GCs were cultured with DMEM/F12 (1:1) medium containing 15% FBS, 1% Insulin-transferrin-Se, and 100 U/ml penicillin and streptomycin at 37°C in a humidified atmosphere with 5% CO_2_. The first passage of GCs was used in all experiments and the ovarian GCs were identified by detecting the density of immunofluorescence for FSHR expression since only ovarian GCs express follicle-stimulating hormone receptors (FSHR) in ovarian tissue.

**Supplementary Figures and Legends**

**
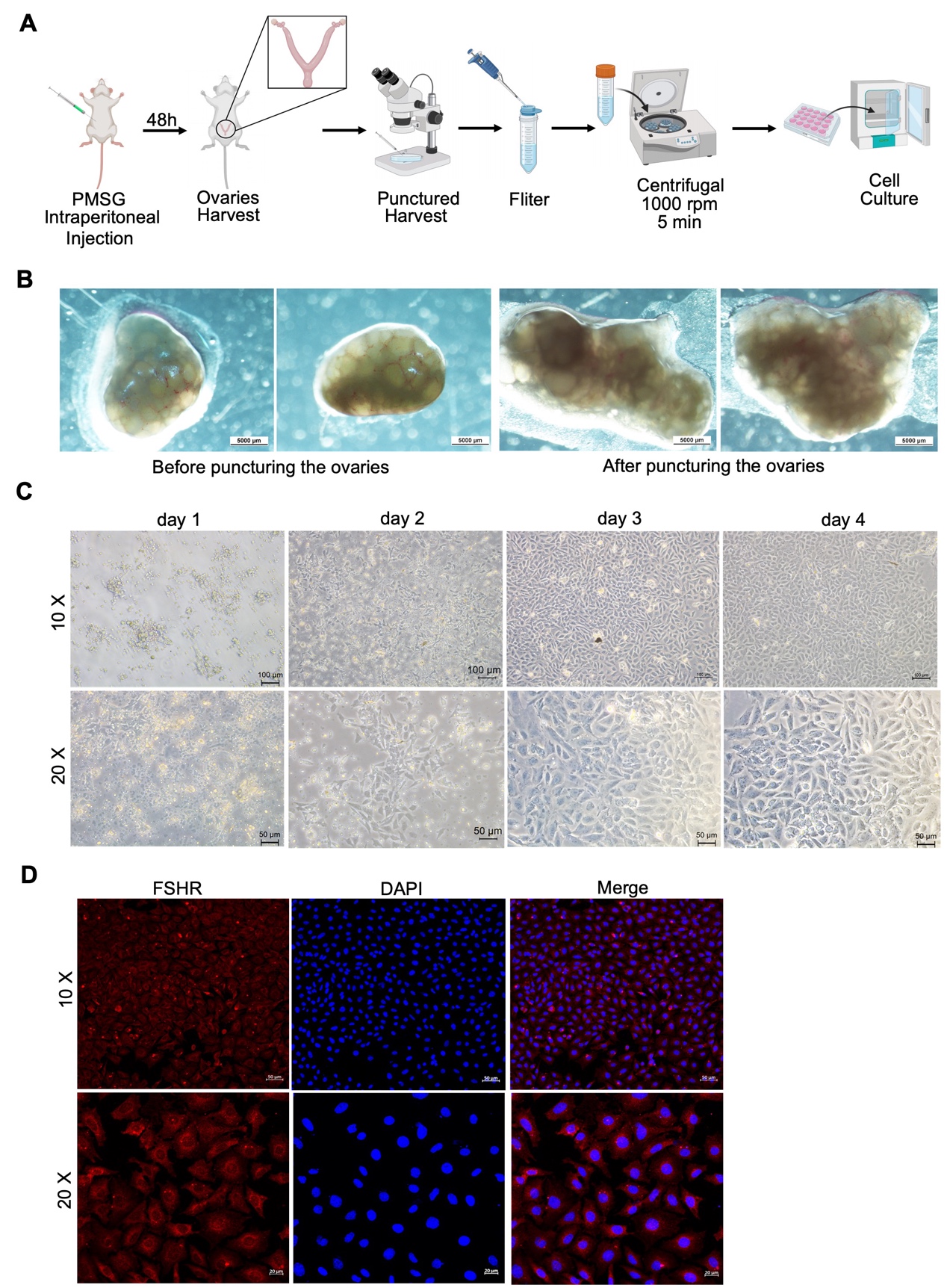
**

**Supplementary Fig 1.** **Extraction and identification analysis of GCs.** (A) Schematic diagram of the extraction of ovarian GCs by mechanical method. (B) After puncturing the follicle under a body microscope, a large amount of follicular fluid flows out. (C) Morphological appearance of cultured GCs. (D) Immunofluorescence staining of the surface markers FSHR in GCs.

**Supplementary Fig 2. Flow cytometry to detect cell death. (A-B) Quantitative analysis of flow cytometry results.**

**
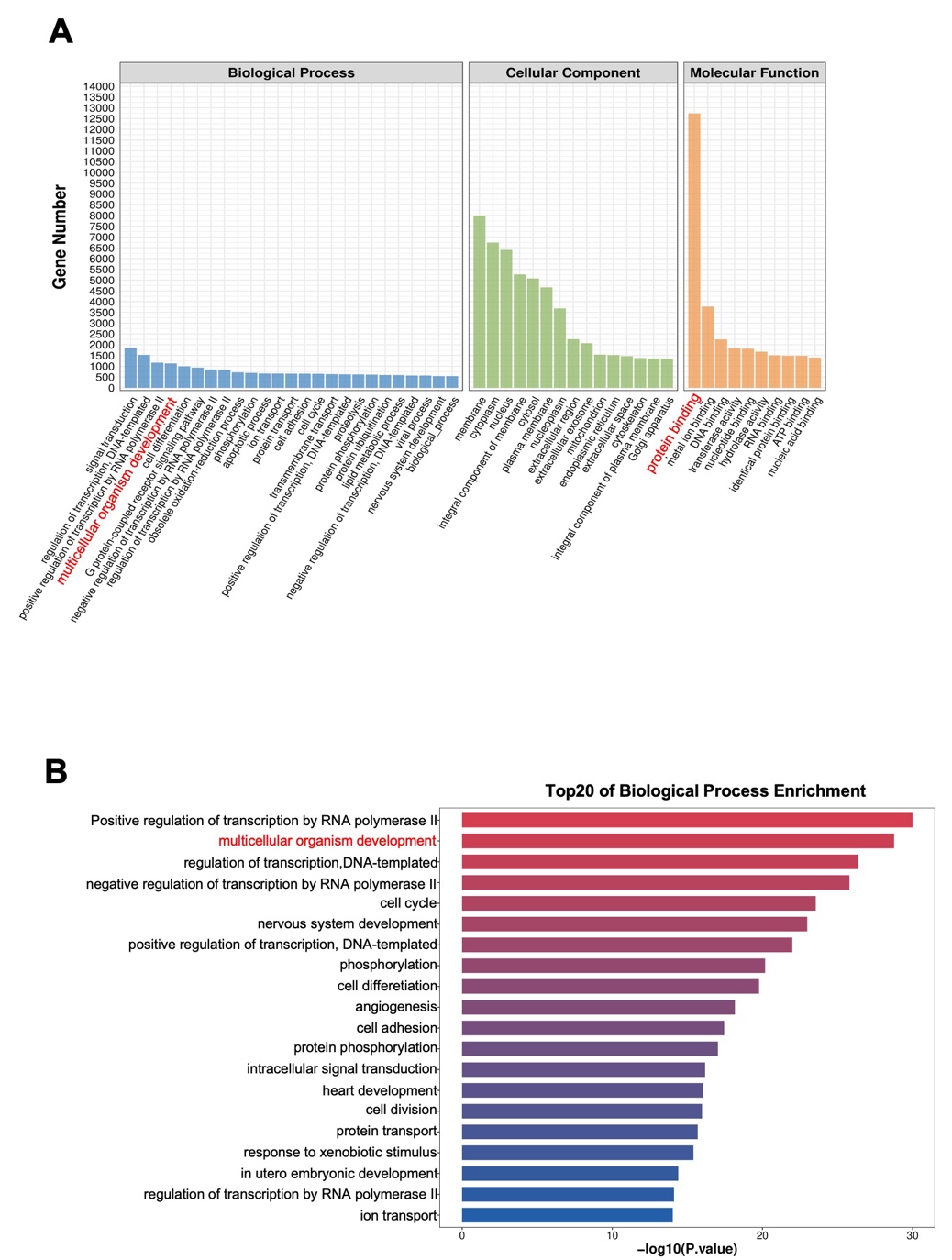
**

**Supplementary Fig 3. Go analysis of miRNA in hUSC-Exo.** (A) GO analysis of the targeted genes. (B) Top 20 of biological process enrichment.

**
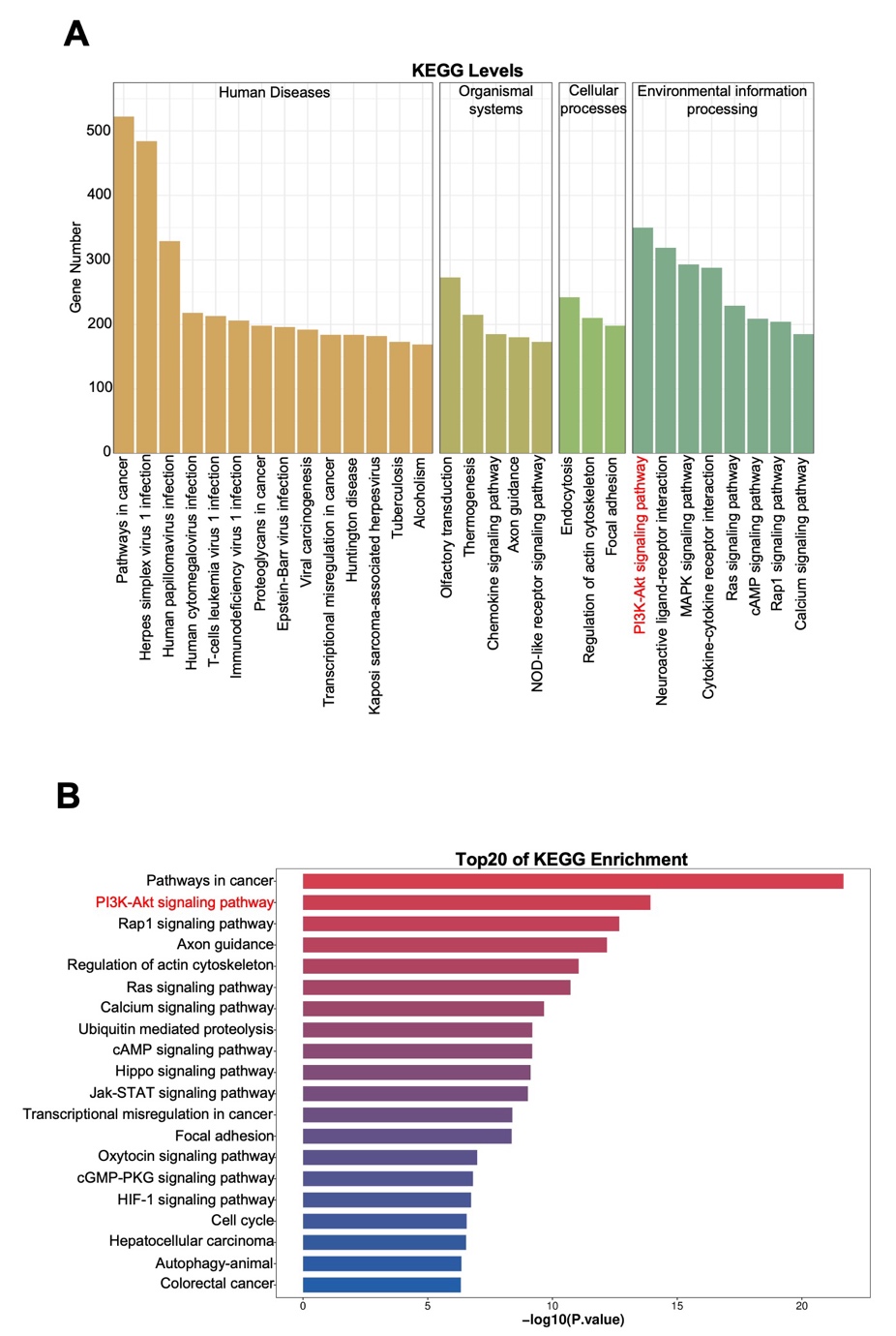
**

**Supplementary Fig 4. KEGG analysis of miRNA in hUSC-Exo.** (A) KEGG level analysis of differentially expressed genes in the hUSC-Exo and DFL-Exo groups. (B) Top 20 KEGG pathways enrichment analysis.

**
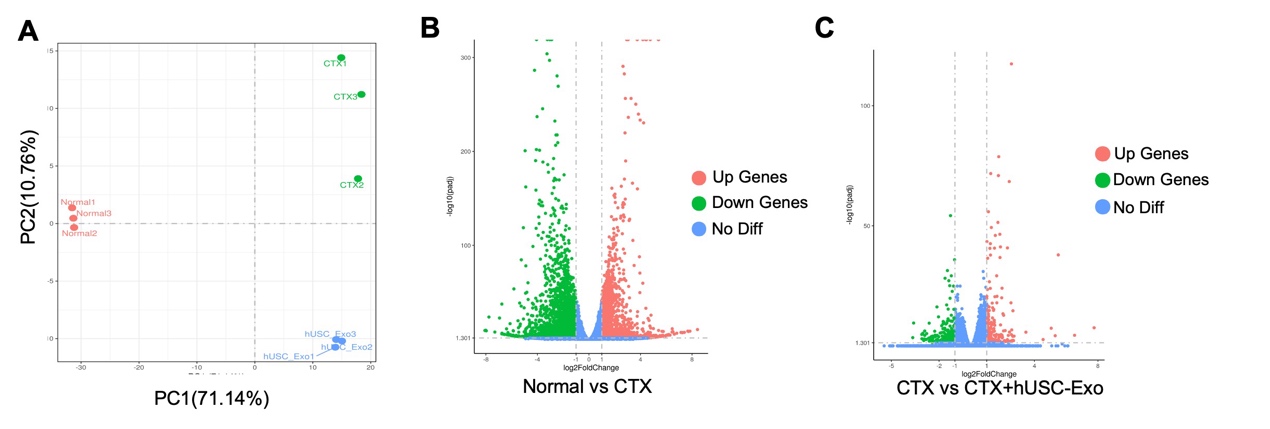
**

**Supplementary Fig 5. Expression of differential genes in GCs.** (A) Principal component analysis in the three groups of samples. (B) The up or down-regulated genes in the CTX group compared with normal group. (C) The up or down-regulated genes in the CTX + hUSC-Exo group compared with the CTX group.

**Supplementary Table1. Primers and conditions used for RT-PCR to detect gene transcripts in hUSC_S_.**

| Genes | Sequence | TM（℃） | Size（bp） |
| --- | --- | --- | --- |
| GAPDH | F:5’-CCACCCATGGCAAATTCCATGGCA-3’ | 59 | 598 |
|  | R:5’-TCTAGACGGCAGGTCAGGTCCACC-3’ |  |  |
| Nanog | F:5’-CAATGGTGTGACGCAGGGAT-3’ | 52 | 249 |
|  | R:5’-TGCACCAGGTCTGAGTGTTC-3’ |  |  |
| OCT4 | F:5’-ATCCCTGAACCTAGTGGGGA-3’ | 59 | 240 |
|  | R:5’-CACTCGGACCACATCCTTCT-3’ |  |  |
| CD105 | F:5’-TCCTCCCAAGGACACTTGTA -3’ | 57 | 244 |
|  | R:5’-CGCCTCATTGCTGATCATAC-3’ |  |  |
| CD29 | F:5’-CCTACTTCTGCACGATGTGATG-3’ | 60.7 | 128 |
|  | R:5’-CCTTTGCTACGGTTGGTTACATT-3’ |  |  |
| CD34 | F:5’-AATCAGCACAGTGTTCACCAC-3’ | 60.5 | 218 |
|  | R:5’-TGCCCTGAGTCAATTTCACTTC-3’ |  |  |
| CD133 | F:5’-ATCCTTTCCATTACGGCGGC-3’ | 57 | 311 |
|  | R:5’-CTCAAGGCACCATCCCGTG-3’ |  |  |
| F, Forward primer; R, Reverse primer; | | | |

**Supplementary Table2. Primer sequences for real-time quantitative PCR to detect related gene transcripts in GCs.**

| Genes | Sequence |
| --- | --- |
| GAPDH | F:5’-CCACATCGCTCAGACACCAT-3’ |
|  | R:5’-GCGCCCAATACGACCAAAT-3’ |
| Heg1 | F:5’-TTCTCAAACAGAGAGTGGGACA-3’ |
|  | R:5’-TTCCTGGCCGGATGAATTTCT-3’ |
| Pdia4 | F:5’-TCCCATTGCTGTAGCGAAGAT-3’ |
|  | R:5’-GGGGTAGCCACTCACATCAAAT-3’ |
| Tmem86a | F:5’-CTGTGGTGAAGAGCGAAGGAC-3’ |
|  | R:5’-GGGCAGGCACTTGATAAGGG-3’ |
| Ankrd | F:5’-GCTGGTAACAGGCAAAAAGAAC-3’ |
|  | R:5’-CCTCTCGCAGTTTCTCGCT-3’ |
| Slc1a4 | F:5’-GGCATCGCTGTTGCTTACTTC-3’ |
|  | R:5’-CGAGGAAAGAGTCCACTGTCT-3’ |

**Supplementary Table 3. The differentially expressed miRNA of the heat map**

| **miRNA** | **hUSC-Exo 1** | **hUSC-Exo 2** | **DFL-Exo 1** | **DFL-Exo 2** |
| --- | --- | --- | --- | --- |
| miR-2904_R+3 | 2445 | 3338 | 71 | 337 |
| miR-221-3p | 3273 | 3086 | 443 | 700 |
| miR-5106_R-4 | 3634 | 413 | 178 | 189 |
| miR-181a-5p | 1496 | 1187 | 403 | 366 |
| miR-27b-3p | 2081 | 2051 | 1539 | 996 |
| miR-451a_R+1 | 743 | 917 | 46 | 47 |
| miR-423-3p | 1106 | 945 | 234 | 407 |
| miR-200b-3p | 476 | 930 | 0 | 16 |
| miR-100-5p | 1293 | 975 | 437 | 459 |
| miR-144-3p | 538 | 736 | 14 | 0 |
| miR-30c-5p | 814 | 908 | 281 | 197 |
| miR-181b  -5p_R+1 | 880 | 676 | 257 | 147 |
| miR-1246_R+1 | 656 | 779 | 120 | 222 |
| miR-148a-3p | 704 | 580 | 148 | 101 |
| miR-2887-1-p3 | 505 | 1035 | 110 | 402 |
| miR-2887-1-p5 | 505 | 1035 | 110 | 402 |
| miR-6240-p3 | 594 | 1065 | 449 | 197 |
| miR-6240-p5 | 594 | 1065 | 449 | 197 |
| miR-339-5p_R+3 | 508 | 521 | 15 | 41 |
| miR-146a-5p | 448 | 499 | 0 | 19 |
| miR-25-3p | 894 | 999 | 654 | 372 |
| miR-5119_L | 252 | 687 | 0 | 79 |
| miR-92a-3p | 620 | 737 | 448 | 236 |
| miR-148b-3p | 424 | 434 | 93 | 114 |
| miR-320a-3p | 1157 | 1084 | 384 | 1277 |
| miR-186-5p_R+1 | 430 | 317 | 95 | 101 |
| miR-193b-3p | 276 | 293 | 10 | 16 |
| miR-423-5p | 482 | 442 | 139 | 243 |
| miR-1246_R+2 | 290 | 314 | 5 | 66 |
| miR-10a-5p_R+1 | 635 | 1136 | 751 | 492 |

**Supplementary Table 4. The differentially expressed genes of the heat map**

| **High expressed in Normal and hUSC-Exo but low expresses in CTX** | | | |
| --- | --- | --- | --- |
| **Genes** | **Mean_Normal** | **Mean_CTX** | **Mean_hUSC-Exo** |
| Heg1 | 46857.47797 | 5730.368029 | 12185.8562 |
| Pdia4 | 15356.43828 | 7489.762254 | 14932.07482 |
| Tmem86a | 14509.41484 | 2293.309864 | 5205.252283 |
| Adamts9 | 7731.769681 | 1042.392423 | 2412.945483 |
| Ankrd1 | 6339.289072 | 1579.856919 | 4602.399232 |
| Masp1 | 4962.856504 | 816.9739871 | 1924.636336 |
| Large1 | 3367.834168 | 659.1330514 | 1308.807299 |
| Smarca1 | 3326.543317 | 876.2358528 | 1777.097595 |
| Tenm4 | 2121.047844 | 145.4925394 | 555.6126377 |
| Tmtc2 | 1600.373118 | 132.875306 | 283.8429253 |
| Vps13b | 1249.30687 | 342.1672164 | 726.1249327 |
| Kif11 | 1109.316855 | 209.6860464 | 441.1784061 |
| Prlr | 1107.453855 | 199.871647 | 436.1126513 |
| Ifi27 | 1044.241127 | 482.7452793 | 1554.782333 |
| Nrp2 | 962.1255822 | 359.1198424 | 724.5225413 |
| Cenpf | 828.3393265 | 129.8302869 | 299.3481863 |
| A730049H05Rik | 795.3792131 | 236.2461764 | 1252.678822 |
| Med16 | 760.3589825 | 152.8844282 | 302.6199202 |
| Prrt4 | 755.8564884 | 106.9435661 | 221.902337 |
| Mturn | 741.0067454 | 204.1429018 | 406.0888645 |
| Scx | 683.889906 | 250.0390369 | 546.8674642 |
| Abcb8 | 598.4375777 | 247.784098 | 527.5840947 |
| Kif2c | 568.0050604 | 81.7403378 | 197.874149 |
| Sumo2 | 560.5492517 | 199.8670769 | 484.8601997 |
| Espl1 | 552.9199698 | 124.6638667 | 299.5853813 |
| Rims4 | 543.0874081 | 244.2732898 | 859.1205781 |
| Adgrv1 | 518.4349733 | 214.1128699 | 445.1330874 |
| Brca1 | 497.2541038 | 136.1765958 | 302.1952496 |
| Itpr2 | 486.1337899 | 196.7714207 | 434.3573926 |
| Kntc1 | 469.9928835 | 94.13944906 | 289.4650818 |
| Bub1 | 452.5805851 | 108.6243944 | 261.1494542 |
| Pbk | 429.0563204 | 69.96102572 | 155.2885385 |
| Sh3tc1 | 426.8508959 | 169.7679219 | 425.44284 |
| Cenpa | 418.1938377 | 207.5658886 | 677.9475506 |
| Pimreg | 398.3174659 | 64.99740567 | 148.2501562 |
| Tmem151a | 369.5758192 | 152.7964025 | 321.3603274 |
| E2f7 | 324.5348488 | 45.47588472 | 102.3831357 |
| Fstl4 | 307.2948939 | 104.1630776 | 247.7600155 |
| Kif15 | 294.4024517 | 45.12194594 | 105.5397297 |
| Kif14 | 292.9189612 | 48.0894419 | 111.7102836 |
| **High expressed in CTX but low expresses in Normal and hUSC-Exo** | | | |
| **Genes** | **Mean_Normal** | **Mean_CTX** | **Mean_hUSC-Exo** |
| mt-Rnr1 | 1210.97459 | 10411.6938 | 3882.84561 |
| mt-Rnr2 | 3445.29342 | 8234.89885 | 3575.47079 |
| Slc1a4 | 669.290313 | 4658.95372 | 1893.05237 |
| Zfp365 | 729.204685 | 4393.46756 | 2035.0723 |
| Jag1 | 1089.84263 | 2358.76816 | 753.858253 |
| Sipa1l2 | 418.834983 | 1192.89289 | 423.956063 |
| Plekhh1 | 283.257086 | 802.875104 | 303.067818 |
| Map3k9 | 169.791841 | 752.423694 | 296.129852 |
| Kif26a | 167.751477 | 637.578459 | 254.132349 |
| Tent5c | 216.234067 | 603.086845 | 289.687191 |
| Prag1 | 115.927255 | 598.446202 | 292.336824 |
| Ptp4a1 | 121.250278 | 536.407615 | 51.8745279 |
| 5430430B14Rik | 85.8005096 | 534.308517 | 261.722755 |
| Irf6 | 172.117076 | 349.995463 | 127.399673 |
| AC125351.1 | 41.5452554 | 253.54238 | 95.0763264 |
| Cmya5 | 60.4821786 | 217.897335 | 25.1477111 |
| Tec | 34.3548994 | 205.529069 | 98.8149778 |
| Gm32443 | 95.8401784 | 196.288534 | 47.281379 |
| Cdcp1 | 54.7608712 | 179.675954 | 71.4110142 |
| Hspa1a | 27.5224863 | 168.854779 | 61.2040749 |
| Ereg | 50.9603613 | 151.607149 | 56.2019276 |
| Trim6 | 15.1768838 | 143.653399 | 124.198433 |
| Tmem158 | 20.951785 | 136.49093 | 40.0037102 |
| Wnt9a | 33.2702188 | 133.738215 | 42.5047422 |
| Sh3d21 | 50.8668226 | 127.974164 | 58.2972795 |
| Acer2 | 39.329246 | 127.224433 | 39.329246 |
| P2rx7 | 22.1480606 | 120.151826 | 55.0979645 |
| Foxq1 | 4.9335043 | 119.43079 | 52.3071448 |
| D6Ertd527e | 5.32611193 | 99.5185119 | 29.5859554 |
| Esam | 12.5542214 | 87.9459977 | 34.4364331 |
| Nuak2 | 8.30492226 | 87.1213123 | 32.8055997 |
| Slc16a9 | 9.59473661 | 84.3632076 | 34.7798446 |
| Snord55 | 13.1857302 | 83.0471105 | 24.196561 |
| Chd5 | 6.9290406 | 82.266202 | 29.3712645 |
| Fam71f2 | 2.93358534 | 78.7764723 | 32.8431896 |
| Shisa2 | 31.2746825 | 76.5596574 | 25.2502185 |
| Gm14547 | 7.90046055 | 66.4855563 | 25.9286752 |
| Gm36989 | 19.477982 | 66.3411369 | 31.3932494 |
| Dhrs9 | 11.6258351 | 65.3510645 | 26.6618993 |
| Ccdc170 | 8.99131871 | 62.2914051 | 19.7410271 |
| Fam196a | 8.58247434 | 60.093798 | 20.2499673 |
